# Genome-Wide Study of MYB Transcription Factors in Maize and Their Essential Roles in Male Fertility and Other Biological Processes

**DOI:** 10.3390/ijms27135822

**Published:** 2026-06-27

**Authors:** Yilin Jiang, Huayang Cai, Yang Yang, Qingping Jiang, Xueli An

**Affiliations:** 1 Key Laboratory for Resource Plants Protection and Utilization of Yili Valley in Xinjiang, College of Biological Sciences and Technology, Yili Normal University, Yining 835000, China; 2 Beijing Key Laboratory of Maize Bio-Breeding, Research Institute of Biology and Agriculture, School of Advanced Agricultural Sciences, University of Science and Technology Beijing, Beijing 100083, China

**Keywords:** MYB transcription factors, maize, phylogenetic analysis, anther and pollen development

## Abstract

MYB transcription factors (TFs) play essential roles in diverse biological processes, including anther and pollen development, vegetative growth, seed development and germination, and stress responses. However, functional characterization of MYB TFs in maize (*Zea mays*) lags far behind that in *Arabidopsis thaliana* and *Oryza sativa*. In this study, we performed a genome-wide identification of 196 maize MYB TFs, along with phylogenetic analysis and Gene Ontology (GO) annotation. To bridge the knowledge gap, we established an integrated cross-species comparative workflow that systematically maps functionally characterized MYB TFs from *Arabidopsis* and rice to their maize orthologs. By coupling this homology-based approach with spatiotemporal expression profiling of developing anthers across multiple inbred lines, we prioritized candidate MYB TFs likely involved in anther and pollen development. This integrated strategy provides a useful reference for translating the rich functional knowledge accumulated in model plants to crops with less-characterized genomes. Our study not only establishes a solid foundation for the functional investigation of maize MYB TFs, but also offers promising targets for the mechanistic dissection and molecular breeding application of male sterility in maize.

## 1. Introduction

Transcription factors (TFs) are a class of essential regulatory proteins that, upon sensing diverse stress signals, become activated and subsequently modulate the expression of stress-responsive genes, thereby enhancing plant survival under adverse conditions [1]. The MYB (v-myb avian myeloblastosis viral oncogene homolog) family constitutes one of the largest and most functionally versatile families of transcription factors in plants. The first identified MYB TF gene, *v-myb*, was originally discovered as an oncogene carried by the avian myeloblastosis virus (AMV), which induces myeloblastosis in chickens. The first plant *MYB* gene was identified in maize (*Zea mays*) [2,3].

Plant MYB TFs are distinguished by highly conserved repetitive sequences (Rs) within the N-terminal MYB DNA-binding domain (DBD) [4]. Each repeat comprises approximately 52 amino acids that fold into three α-helices. In this structural configuration, the second and third α-helices form a typical helix-turn-helix (HTH) structure, while three evenly distributed tryptophan or other hydrophobic residues constitute the hydrophobic core [5]. The third α-helix functions as the primary recognition helix to bind the major groove of target DNA molecules. Due to structural homology with the c-Myb protein, these repetitive segments were renamed R1, R2, and R3. According to the number of adjacent repeats (R) in the MYB domain, four distinct subgroups of MYB structural variants have been identified, namely 1R-MYB, R2R3-MYB, 3R-MYB, and 4R-MYB. Most plant MYB proteins contain two conserved repeats and are classified into the R2R3-MYB subfamily [6]. Unlike the N-terminal conserved domain, the C-terminal region exhibits diverse regulatory functions. Specifically, the C-terminal EAR repression motif and TLLLFR repression motif are responsible for negative transcriptional regulation. By contrast, the C-terminal SG6 and SG7 motifs cooperatively mediate positive regulatory functions. Moreover, conserved serine and threonine residues are distributed in the C-terminal region and are predicted to serve as potential post-translational modification sites [7].

From an evolutionary perspective, the initial diversification of the MYB superfamily tracks back to early eukaryotic evolution, with a significant expansion and functional divergence of the R2R3-MYB subfamily occurring during the transition of plants from aquatic environments to land (approximately 400–500 million years ago) [8,9]. This macro-evolutionary expansion equipped land plants with sophisticated regulatory networks to adapt to terrestrial hardships, such as desiccation and UV radiation.

MYB transcription factors (TFs) regulate diverse biological processes, including anther and pollen development, vegetative and seed development, biotic and abiotic stress responses, and plant secondary metabolism [10,11]. Current research has primarily focused on their roles in environmental stress, morphogenesis, and metabolite biosynthesis. Among these, MYB TFs are particularly well characterized as regulators of phenylpropanoid metabolism, e.g., in the biosynthesis of proanthocyanidins, anthocyanins, flavonols, and lignin [12,13].

In the model plants *Oryza sativa* and *Arabidopsis thaliana*, the *MYB* gene family comprises 233 and 251 members, respectively [14]; numerous members have been functionally characterized and are involved in the extensive processes mentioned above, including reproductive development, stress responses, and secondary metabolism. Specifically, 17 MYB TFs are involved in pollen development and male fertility regulation, and MYB proteins perform vital roles in the tapetum, extracellular space, and microspores. For example, microspores of heterozygous *myb81-1* mutants fail to complete pollen mitosis I (PMI) and become arrested at the polarization stage with a single central vacuole. Pollen development is blocked at the polarized microspore stage, resulting in defective asymmetric cell division during PMI [15]. *DUO1* modulates the activity of the G2/M checkpoint in the cell cycle and facilitates germ cell division by activating downstream targets such as cyclin genes. Mutation of *DUO1* prevents germ cells from entering mitosis [16]. Furthermore, *OsGAMYB* is involved in the regulation of nearly all gibberellin-responsive gene expression in anthers. Mutation of *OsGAMYB* results in abnormal programmed cell death (PCD) of tapetal cells, as well as defective development and formation of pollen exine and Ubisch bodies [17].

However, among the 196 MYB TFs identified in maize, only four (*ZmMs9*, *ZmMYB84*, *ZmMYB33-1* and *ZmMYB33-2*) have been reported to be essential for anther development and male fertility [18]. Given the functional conservation of homologous MYB TFs among *A. thaliana*, *O. sativa*, and *Z. mays*, functional studies of MYB TFs in *Arabidopsis* and rice will provide valuable clues for exploring their orthologs in maize.

## 2. Results

### 2.1. The Nomenclature and Characterization of Maize MYB TFs

**Figure 1:**
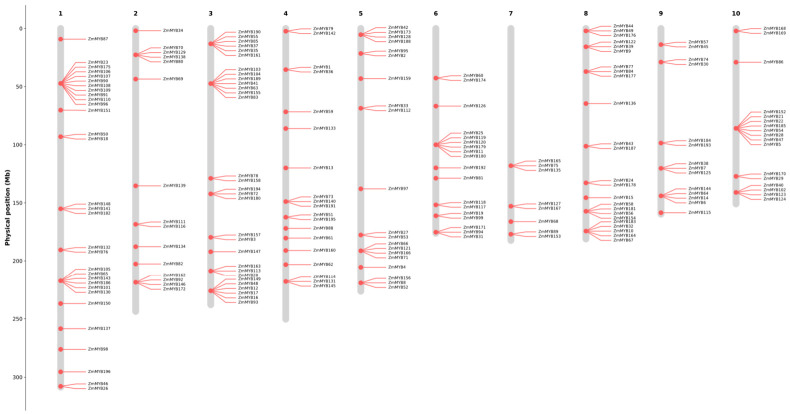
Chromosomal distribution of the 196 ZmMYB TFs on maize chromosomes. The physical locations of 196 maize ZmMYB TFs are mapped across the 10 maize chromosomes. Vertical grey bars represent the chromosomes, with their lengths in megabases (Mb) indicated on the left. Red dots indicate the specific physical positions of the genes on the chromosomes, with the protruding red lines and branches on the right pointing to the specific ZmMYB TFs located within those regions.

Maize MYB TFs can be classified into four categories: 1R-MYB, R2R3-MYB, 3R-MYB, and 4R-MYB, among which most members contain two MYB repeat domains and belong to the R2R3-MYB subfamily [6,19]. Based on the updated maize B73 V5 reference genome (Zm-B73-REFERENCE-NAM-5.0), 196 maize MYB TFs (ZmMYBs) were systematically identified from the MaizeGDB database (access date: 20 March 2026) in this study. To investigate the chromosomal distribution and genomic clustering of *ZmMYB* genes, we mapped their physical positions across the ten maize chromosomes (Figure 1). The results demonstrated that the 196 ZmMYB family members are widely yet unevenly distributed across all chromosomes. Chromosome 1 (Chr 1) harbored the largest number of genes, featuring a prominent gene cluster in its upper-middle region. Concurrently, distinct regional enrichments and hotspots were observed on specific genomic locations of Chr 3, Chr 5, Chr 6, and Chr 10. Notably, multiple *ZmMYB* loci exhibited tightly clustered, multi-branched patterns at adjacent chromosomal positions (e.g., on Chr 1 and Chr 6), strongly implying that tandem duplication events served as a primary driving force for the expansion and evolutionary divergence of this gene family in maize. The specific physical coordinates and gene IDs for each locus are provided in Table S1.

**Figure 2:**
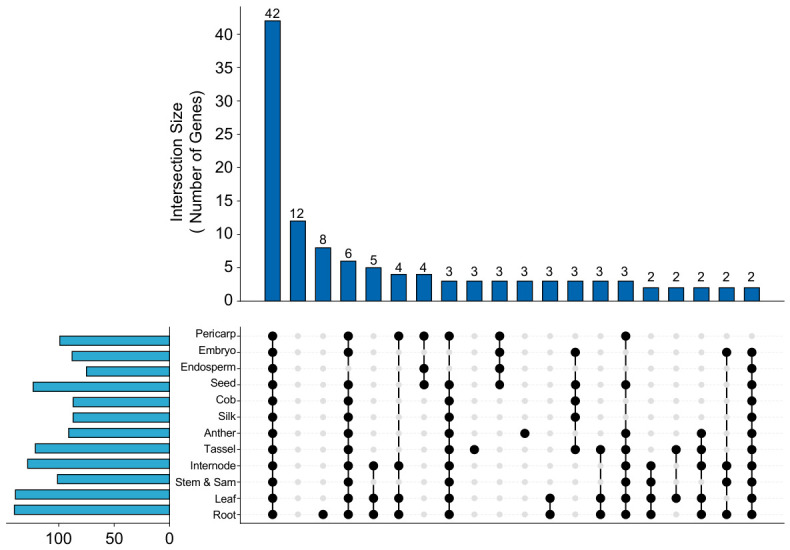
Global expression landscape of the maize MYB transcription factor family. UpSet plot depicting the expression pattern intersections of 196 MYB TFs across 12 tissues. Left horizontal bar chart: total number of MYB TFs expressed in each tissue (Set Size). Top vertical bar chart: number of genes in each intersection (Intersection Size). Bottom dot matrix: tissue composition of each intersection; connected black dots indicate expression in the corresponding tissues, while light gray dots indicate absence. Genes expressed in all 12 tissues are considered constitutive, whereas those restricted to single dots are tissue-specific. Detailed expression information for each gene is provided in Table S1.

In addition, using RNA-seq data from the MaizeGDB database, we systematically analyzed the spatial expression patterns of the 196 identified ZmMYB TFs (Table S1). To investigate their spatio-temporal expression profiles, we examined the presence of these genes across 12 representative tissues (root, leaf, stem/SAM, internode, tassel, anther, silk, cob, seed, endosperm, embryo, and pericarp). UpSet analysis revealed a high degree of functional diversity and specialization within the family (Figure 2). Approximately 21.4% (42/196) of the MYB TFs exhibited a constitutive expression pattern, being detected in all 12 tissues, suggesting their fundamental roles in maize growth and development. In contrast, a substantial number of genes showed distinct tissue-specific or organ-preferential expression. Notably, root and leaf tissues possessed the largest sets of expressed MYB TFs, indicating active transcriptional regulation in vegetative organs. Furthermore, several MYB members were exclusively expressed in reproductive tissues, such as anther and tassel, implying their potential involvement in male reproductive development and lipid metabolism regulation—a finding consistent with the known roles of MYB factors in anther development. The diverse intersection patterns highlight the complex regulatory network of the MYB family in coordinating maize physiological processes.

### 2.2. Phylogenetic Analysis, Classification, and Structural Characterization of MYBs in Maize

**Figure 3:**
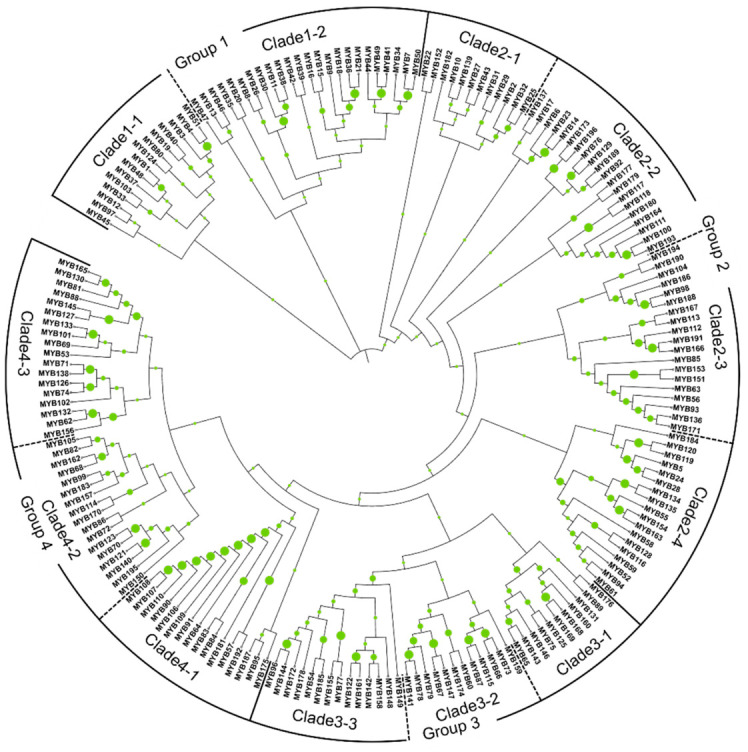
Phylogenetic analysis of the 196 maize MYB TFs. The tree divides the proteins into four major clades (Clade 1–4). Clade 1 contains 39 MYBs and is further divided into two subclades (1-1 and 1-2). Clade 2 contains 69 MYBs and is divided into four subclades (2-1 to 2-4). Clade 3 contains 37 MYBs and is divided into three subclades (3-1 to 3-3). Clade 4 contains 51 MYBs and is divided into three subclades (4-1 to 4-3). The sizes of the green dots on the nodes are proportional to bootstrap support values.

To analyze the phylogenetic relationships of the *MYB* gene family, we constructed a maximum likelihood phylogenetic tree using 196 maize MYB protein sequences and MEGA 11 software (Version 11.0.13). All identified MYBs can be divided into four major clades (Clade 1 to Clade 4) (Figure 3). Clade 1 contains 39 MYBs and is further divided into two subclades (Subclades 1-1 and 1-2). Clade 2 contains 69 MYBs and is divided into four subclades (Subclades 2-1 to 2-4). Clade 3 comprises 37 MYBs and is divided into three subclades (Subclades 3-1 to 3-3). Clade 4 comprises 51 MYBs and is divided into three subclades (Subclades 4-1 to 4-3).

Among them, *ZmMYB33-1* (*MYB67*) and *ZmMYB33-2* (*MYB147*), located in Subclade 3-2, have been reported to be involved in anther development. Mutants of these genes exhibit complete male sterility with smaller anthers and no visible pollen grains. Scanning electron microscopy (SEM) reveals that the inner and outer surfaces of the mutant anther wall are smooth and shiny, indicating a complete loss of function [19]. *OsGAMYB* and *AtMYB33/65* are homologs of *ZmMYB33*. *OsGAMYB* is involved in anther development and is regulated by miRNAs in anthers. Additionally, *OsGAMY*B and its upstream regulator SLR1 participate in gibberellin (GA)-mediated gene expression in rice grains, acting as bifunctional genes [20]. In *Arabidopsis*, the anther development of *atmyb33 atmyb35* double mutants is also defective; the tapetum at the pollen mother cell stage becomes hypertrophic, eventually leading to incomplete pollen development during Prophase I, and *AtMYB33* is also regulated by miRNAs [21]. Notably, *ZmMYB33-1* (*MYB67*) *and ZmMYB33-2* (*MYB147*) are expressed across a wide range of maize tissues (Figure 2; Table S1), suggesting that they may participate in biological processes beyond anther development.

Two MYBs in Clade 4 are involved in pollen or anther development. *ZmMs9* (*MYB175*) is located in Subclade 4-1 and is involved in anther development. In *zmms9* mutants, after tapetal cells complete mitosis, abnormal parenchyma structures frequently form between the two nuclei, eventually causing microspore abortion. *Arabidopsis TDF1* and rice *OsTDF1* are orthologs of *ZmMs9* [22]. *AtTDF1* encodes an R2R3-MYB transcription factor involved in anther development and is highly expressed in the tapetum, meiotic cells, and microspores. Its mutants exhibit disordered tapetal division and functional defects, eventually leading to microspore abortion [23]. *OsTDF1* is also involved in anther development; *ostdf1* knockout mutants show complete male sterility, and their tapetal cells display typical vacuolation and abnormal hypertrophy, highly similar to *attdf1* mutants. Heterologous expression of *OsTDF1* in *attdf1* mutants fully restores fertility, demonstrating that this homologous gene can complement the physiological function of *TDF1* in *Arabidopsis*. *AtTDF1* and *OsTDF1* may also complement the function of *ZmMs9* in maize [24].

*ZmMYB84* (*MYB170*) is located in Subclade 4-2. Homozygous *myb84* mutants exhibit complete male sterility, with normal vegetative organ growth and normal female fertility. Compared with wild-type anthers, *myb84* anthers possess smaller pollen grains, a denser outer epidermis of the anther wall, and a smooth inner surface due to the absence of Ubisch bodies, ultimately leading to male sterility [19]. *OsMYB80* and *AtMYB80* are homologous genes of *ZmMYB84*. *OsMYB80* TF is involved in the regulation of anther development and is barely expressed in other tissues except anther tissue. This gene starts to be expressed at the anther meiosis stage, reaches the expression peak at the tetrad release stage, and then its expression level decreases rapidly. *osmyb80* mutants exhibit typical defective phenotypes such as PCD of tapetum, lack of ubisch bodies, failure of normal pollen exine formation, and microspore abortion, eventually leading to microspore abortion [25]. *AtMYB80* (originally named *AtMYB103*) is involved in the regulation of anther development and is a key gene regulating tapetum and pollen development. *atmyb80* mutant shows abnormal tapetum development and disordered callose degradation; most microspores in mature anthers are degraded, and the remaining microspores lack the pollen exine structure [26,27]. Furthermore, both *ZmMs9* (*MYB175*) and *ZmMYB84* (*MYB170*) are expressed in maize tassels, with *ZmMs9* (*MYB175*) showing relatively tassel-specific expression, implying a predominant role in male reproductive development.

Both *ZmMs9* and *ZmMYB84* are located in Clade 4, indicating that they originated from the same ancestral R2R3-MYB gene, belong to the same evolutionary lineage, and retain the conserved MYB DNA-binding domain, thus both possess the molecular basis for regulating anther development. Their location in different subclades is most likely due to gene duplication events. After duplication, the two copies accumulated distinct mutations, progressively reducing sequence homology and eventually forming independent evolutionary subclades with divergent regulatory functions: *ZmMs9* is essential for normal tapetal cell development, whereas *ZmMYB84* contributes to pollen wall formation and Ubisch body deposition. By contrast, *ZmMYB33-1/-2* are located in Clade 3, representing an independent evolutionary lineage whose ancestral gene diverged early from the Clade 4 ancestors with substantial sequence differences, and they primarily regulate anther size. Collectively, the phylogenetic analysis not only reveals the evolutionary relationships of *MYB* TFs, but also provides valuable clues for exploring the functions of uncharacterized MYB TFs in maize.

To structurally and functionally characterize the MYB transcription factor family in maize, we systematically analyzed their gene organizations, upstream promoter architectures, and protein conserved domains. Transcript structural analysis revealed a diverse yet lineage-specific exon-intron distribution across the ZmMYB members, where distinct configurations of coding sequences (CDS) and untranslated regions (UTRs) underscore structural divergence during genomic evolution (Figure S1). To further elucidate the transcriptional regulation of these genes in response to internal and external stimuli, a 3000 bp upstream promoter analysis was conducted. This revealed a highly enriched and distinct repertoire of cis-acting regulatory elements, notably those involved in light responsiveness, anaerobic induction, and zein metabolism regulation, suggesting that *ZmMYB* genes are heavily involved in complex environmental adaptation and developmental networks (Figure S2). Furthermore, conserved domain analysis (CDD) confirmed the structural integrity of the typical MYB DNA-binding domains across the identified sequences, illustrating that despite significant divergence in their non-coding and promoter regions, their core biochemical functionality and DNA-binding modules remain highly conserved throughout evolution (Figure S3). Taken together, these comprehensive architectural profiles provide crucial insights into the evolutionary dynamics of the maize MYB family and establish a structural foundation for selecting key candidate genes involved in stress tolerance and metabolic regulation for future functional validation.

### 2.3. Gene Ontology Analysis of MYBs in Maize

**Figure 4:**
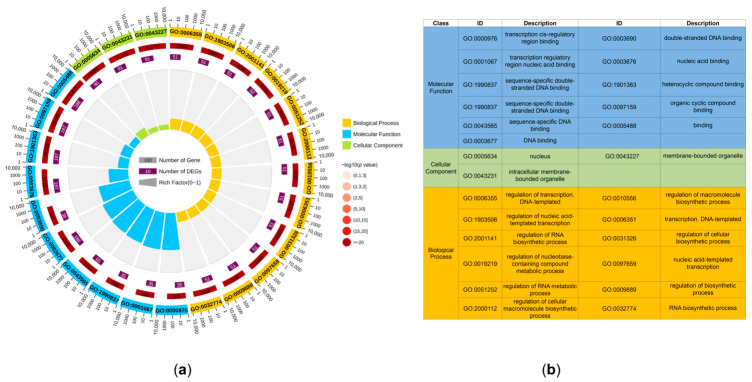
Gene Ontology (GO) enrichment analysis of maize MYB TFs. (**a**) GO enrichment results, showing the rich factor, the number of associated genes, and the *p* value for each significantly enriched GO term. First circle (outermost): Displays enriched GO terms categorized by color (Biological Process, Cellular Component, Molecular Function), with the outer scale indicating gene counts. Second circle: Shows background gene numbers and statistical significance. Bar lengths are proportional to gene counts, and color intensity (blue to red) reflects *p* values (deeper red indicates higher significance). Third circle: Presents differentially expressed (DE) genes within each category, showing the specific counts or ratios. Fourth circle (innermost): Displays the Rich Factor (foreground/background gene ratio) for each category, with grid lines spaced at 0.1 intervals for visual estimation. (**b**) IDs and descriptions of the enriched GO terms.

In molecular biology and bioinformatics, the most widely used structured vocabulary for functional annotation is Gene Ontology (GO). The GO database is commonly employed for the functional annotation of gene products and for the enrichment analysis of large-scale gene or protein datasets generated by omics experiments [28]. To decipher the molecular functions and putative pathways involving maize MYB TFs, we performed GO enrichment analysis. A total of 196 ZmMYBs were annotated to 26 significantly enriched GO terms (Figure 4a). The analysis reveals that these ZmMYBs can be classified into the three major GO categories: molecular function, cellular component, and biological process, which contain 11, 3, and 12 significant terms, respectively (Figure 4b). All 26 GO terms could be further grouped into three functional clusters: DNA binding and transcriptional regulation (11 terms), intracellular localization (3 terms), and transcriptional regulation and biosynthesis (12 terms). Notably, approximately 187 ZmMYBs are involved in DNA binding, and 51 ZmMYBs participate in RNA synthesis and transcriptional regulation, which is consistent with their roles as transcription factors and underscores their importance in gene regulation. 90 ZmMYBs are associated with nuclear localization (Figure 4a). Overall, GO analysis indicates that the *ZmMYB* gene set is mainly enriched in molecular functions such as DNA binding, nucleic acid binding, and sequence-specific DNA binding, is predominantly localized in the nucleus, and primarily functions in biological processes such as transcriptional regulation, RNA biosynthesis, and regulation of gene expression. Thus, ZmMYB TFs encode transcription factors that play key regulatory roles in gene transcription within the nucleus.

### 2.4. Multiple Functions of MYB Transcription Factors in Arabidopsis, Rice and Maize

**Figure 5:**
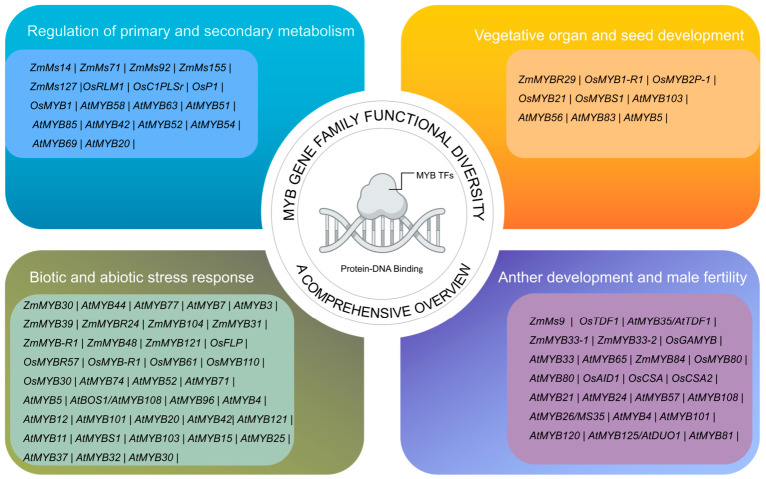
The main functions of MYB TFs in *Arabidopsis*, rice, and maize. Different colors represent distinct biological functional categories: anther and pollen development, vegetative and seed development, biotic and abiotic stress responses, and the regulation of primary and secondary metabolism.

To date, at least 51 and 20 key MYB TFs have been identified in *A. thaliana* and *O. sativa*, respectively, among which 18 are notable in maize (Table S2). Given the functional conservation of orthologous genes among plant species, the characterized functions of MYB TFs in *Arabidopsis* and rice provide important clues for exploring the roles of their orthologs in maize. Accumulating evidence indicates that plant MYB TFs play crucial roles in anther and pollen development, vegetative and seed development, biotic and abiotic stress responses, and the regulation of primary and secondary metabolism (Figure 5 and Table S2).

#### 2.4.1. Anther and Pollen Development

The male reproductive development of flowering plants can be divided into three phases: stamen development, pollination, and fertilization. Two key events occur during stamen development: (1) formation and maturation of pollen grains; (2) filament elongation and anther dehiscence to release mature pollen. During pollination, pollen tubes germinate and deliver sperm cells into the embryo sac to complete double fertilization [11]. MYB TFs, especially R2R3-MYB TFs, have been reported to play important regulatory roles in these complex processes (Table S2).

To date, at least 24 MYB TFs have been reported to be involved in anther and/or pollen development in *A. thaliana*, *O. sativa*, and maize (Table S2 and Figure 5). *AtMYB81* encodes a microspore-specific MYB TF; the *myb81-1* mutant produces approximately 50% abnormal pollen. In this mutant, microspores fail to complete PMI and instead arrest at the polarization stage with a single large central vacuole, while only a small number of microspores are able to divide at later developmental stages [15]. *DUO1* (*AtMYB125*) is specifically expressed in male germ cells from the end of PMI to sperm cell formation; its loss of function results in the production of only a single sperm cell per plant, ultimately causing male sterility [16]. *DUO1* regulates sperm cell specialization by directly activating germline-specific genes such as *MGH3*, *GEX2*, and *GCS1/HAP2* [29,30]. Phylogenetic analysis reveals that *DUO1* expression is positively regulated in a feedback manner by the ROD1 *cis*-element, promoting sperm cell differentiation, and that its function plays a conserved and key role in the reproductive evolution of land plants [31]. Both *AtMYB4* and *AtMYB3*2 maintain pollen wall stability by balancing the metabolic flux of the phenylpropanoid pathway, thereby ensuring normal pollen development. Mutations in both genes result in insufficient flavonoids, abnormal lignin accumulation, and male sterility, characterized by damaged pollen wall structure and collapsed pollen [32,33]. *OsCSA* is mainly expressed in the anther tapetum and vascular tissues; the *oscsa* mutant exhibits complete pollen sterility, with delayed degradation of anther wall cell layers and blocked pollen maturation. *OsCSA2* is specifically expressed in anthers and sugar transport-related vascular tissues; it confers fertility under short-day conditions but shows partial male sterility under long-day conditions, opposite to *OsCSA*. ^14^C-labeled sugar experiments confirmed that the capacity of anther sink tissues to accumulate sugars is significantly decreased in *oscsa* mutants. *OsCSA* controls the distribution of photosynthates to anthers by regulating sugar transport and metabolism genes such as *MST8*, *INV4*, and *SUT3*, and its expression is regulated by photoperiod and the brassinosteroid regulator BZR1 [34,35,36,37]. *AtMYB21*, *AtMYB24*, and *AtMYB57* are functionally redundant R2R3-MYB TFs that are hierarchically regulated by gibberellin (GA) and jasmonic acid (JA), with *AtMYB21* acting as the major effector; together they regulate filament elongation and anther dehiscence. The three proteins can form a complex with bHLH proteins and modulate pollen phenylpropanoid metabolism through the *AtMYB99*–*MYB21*–*MYB24* ternary module. *AtMYB108* is also regulated by JA and cooperates with *AtMYB24* to control anther dehiscence, filament elongation, and pollen viability, forming part of the core regulatory network for stamen maturation [38,39,40]. *AtMYB26/MS35* directly activates *NST1/NST2* to regulate endothecium secondary wall thickening and lignin synthesis. Its expression is directly induced by ARF8.4, and loss of function leads to failure of anther dehiscence and pollen release, ultimately causing male sterility [41,42,43]. *AID1* is a 1R-MYB TF; the sterility of the anther indehiscent mutant *aid1* is mainly caused by defective anther dehiscence, and *AID1* is also involved in programmed cell death (PCD) during anther dehiscence and secondary metabolite deposition during pollen maturation. *AtMYB97*, *AtMYB101*, and *AtMYB120* are pollen tube-specific, with highly similar sequences and functional redundancy. The *myb97*/*myb101*/*myb120* triple mutant shows no abnormalities in pollen development, pollen germination, pollen tube elongation, or pollen tube guidance; however, after the pollen tube enters the embryo sac, it exhibits disordered and continuous growth and fails to release sperm cells normally [44].

These results indicate that MYB TFs regulate processes such as pollen grain formation and maturation, anther dehiscence, filament elongation, and pollen tube recognition. They exert their functions by controlling target gene expression, metabolic pathway balance, and protein–protein interactions, some of which are under hormonal and photoperiodic regulation. Loss of function generally leads to male sterility, highlighting a complex regulatory network in which MYB TFs play key roles during male reproductive development in flowering plants.

#### 2.4.2. Vegetative and Female Organ Development

Another important function of MYB TFs relates to vegetative growth and seed development (Table S2 and Figure 5). Previous studies have shown that various MYB TFs participate in vegetative organ development. For example, *AtMYB103* is expressed not only in the anther tapetum but also in trichomes. In transgenic lines with reduced *AtMYB103* transcript levels, trichomes on cauline leaves and rosette leaves develop additional branches [45].

In addition, some MYB TFs are involved in seed development or seed germination (Figure 5). *AtMYB56* affects seed development through the maternal pathway; during seed development, the seed coat endothelial cells of *atmyb56* mutants are smaller and contracted, and the number of outer integument cells is reduced [46]. *ZmMYBR2* regulates the expression of grain development-related genes and controls maize grain weight by affecting the grain filling rate [47]. Both *AtMYB5* and *AtTT2* regulate outer seed coat development, with *AtMYB5* playing a dominant role. *AtMYB5* exerts a weak pleiotropic effect on trichome development and tannin synthesis; in *atmyb5* mutants, the downstream regulators of seed coat development, including *TT8*, *GL2*, and *TTG2*, are all down-regulated [48]. In summary, MYB TFs are widely involved in vegetative organ development and seed development, regulating traits such as trichome branching, seed coat cell formation, grain filling, and grain weight through complex transcriptional regulatory networks, and are thus crucial for plant growth and development.

#### 2.4.3. Biotic and Abiotic Stress Response

Upon biotic stress, plants rapidly initiate early signaling events such as reactive oxygen species (ROS) burst and elevation of cytoplasmic calcium concentration, thereby activating hormone regulatory networks and inducing the synthesis of defense-related compounds to establish resistance. MYB TFs participate in disease resistance responses through diverse mechanisms, including regulation of early signal transduction, defense product synthesis, and hormone pathways, with crosstalk among these regulatory routes. Meanwhile, abiotic stresses such as nutrient deficiency, toxic ion excess, temperature extremes, and water deficit can severely inhibit plant growth; the transcriptional regulation of stress-related genes is a central mechanism by which plants establish abiotic stress tolerance [1,49].

Regarding abiotic stress, *ZmMYB30* enhances salt tolerance when ectopically expressed in *A. thaliana* [50]. *AtMYB7* modulates the balance of major ultraviolet (UV) protective compounds by inhibiting multiple genes in the flavonoid pathway, and also affects seed responses to high salinity and abscisic acid (ABA) stress by negatively regulating the ABA signaling pathway [51,52]. Phosphorylated *AtMYB42* can directly activate the expression of *SOS2*, a core salt-stress gene, and positively regulate the salt stress response in *A. thaliana*. *AtMYB42* is also induced by ABA and can regulate stomatal closure as well as osmotic and salt stress tolerance, functioning as an important component of the ABA signaling pathway [53,54]. The methylation level of the *AtMYB74* promoter dynamically changes in response to salt stress, participating in the regulation of salt stress sensitivity during seed germination [55]. *ZmMYBR24* responds to salt, alkali, and low-temperature stresses by regulating the flavonoid biosynthesis pathway [56]. *ZmMYB104* directly binds to and activates *ZmCAT2* transcription, positively regulating maize thermotolerance by enhancing ROS scavenging and alleviating oxidative damage [57]. Expression of *AtMYB11*, *AtMYB12*, and *AtMYB111* is positively regulated by the CRY1–HY5 signaling module in *A. thaliana*, promoting flavonol accumulation in guard cells and maintaining ROS balance [58] (Table S2).

Concerning biotic stress, *AtBOS1* is not only involved in defense against necrotrophic fungi but also positively regulates tolerance to abiotic stresses such as salt, drought, and oxidative stress, making it a key gene for cross-stress responses [59]. The elicitor protein HrpN (Ea) enhances *AtMYB4* expression through the ethylene signaling pathway and positively mediates aphid defense responses by regulating the downstream defense gene *PDF1.2* [60]. ABA signaling mediated by *AtMYB96* enhances plant disease resistance by promoting salicylic acid (SA) biosynthesis [61]. *OsMYB30*, *OsMYB55*, and *OsMYB110* promote ferulic acid accumulation by activating the cinnamic acid or lignin monomer synthesis pathway, thereby improving broad-spectrum resistance to fungi and bacteria [62]. *OsMYB30* positively regulates key lignin synthesis genes, promotes lignin accumulation and cell wall thickening, and synergistically enhances rice resistance to *Magnaporthe oryzae* in the *bsr-d1* pathway [63]. Furthermore, *OsMYB30* positively regulates the expression of *OsPAL6*/*OsPAL8*, promotes SA and lignin synthesis, and positively regulates rice brown planthopper (BPH) resistance through the phenylpropanoid metabolic pathway [64] (Table S2). In summary, *MYB* TFs participate in biotic stress responses by modulating early signals, defense products, and hormone pathways, and endow plants with abiotic stress tolerance by affecting antioxidant defense, osmotic balance, and secondary metabolism, playing key regulatory roles in stress adaptation.

#### 2.4.4. Regulation of Plant Secondary Metabolism

Many MYB TFs are involved in the regulation of secondary metabolism, particularly phenylpropanoid metabolism, cell fate determination, root organogenesis, and reproductive processes. For example, *AtMYB58* and *AtMYB63* act downstream of *SND1* and *MYB46*, specifically activating key lignin synthesis genes and positively regulating secondary cell wall development and lignin deposition in *A. thaliana* [65]. *AtMYB5* specifically regulates the synthesis of indolic glucosinolates, is induced by mechanical and biotic stresses, and can enhance insect resistance [66]. Secondary wall-associated *NAC* domain protein *SND1* and its homologs *NST1/2* and *VND6/7* exert dominant repression on *AtMYB103*, *AtMYB85*, *AtMYB52*, and *AtMYB54* through a hierarchical transcriptional regulatory network, leading to a significant reduction in secondary wall thickening of fiber cells. Overexpression of *AtMYB103* promotes secondary wall thickening in fiber cells, whereas overexpression of *AtMYB85* leads to ectopic lignin deposition in stem epidermal and cortical cells [67]. OsC1 possesses transcriptional activation activity and regulates anthocyanin synthesis in rice leaf sheaths and the formation of the purple phenotype by positively controlling the expression of key anthocyanin biosynthesis genes such as *ANS*, *DFR*, *F3H*, and *F3′H* [68] (Table S2). In summary, distinct MYB TFs mediate the synthesis of secondary metabolites, including lignin, glucosinolates, and anthocyanins, through hierarchical transcriptional regulation, and are broadly involved in cell wall development, insect defense, and morphological trait formation.

### 2.5. Functional Predictions of MYB TFs in Maize

**Figure 6:**
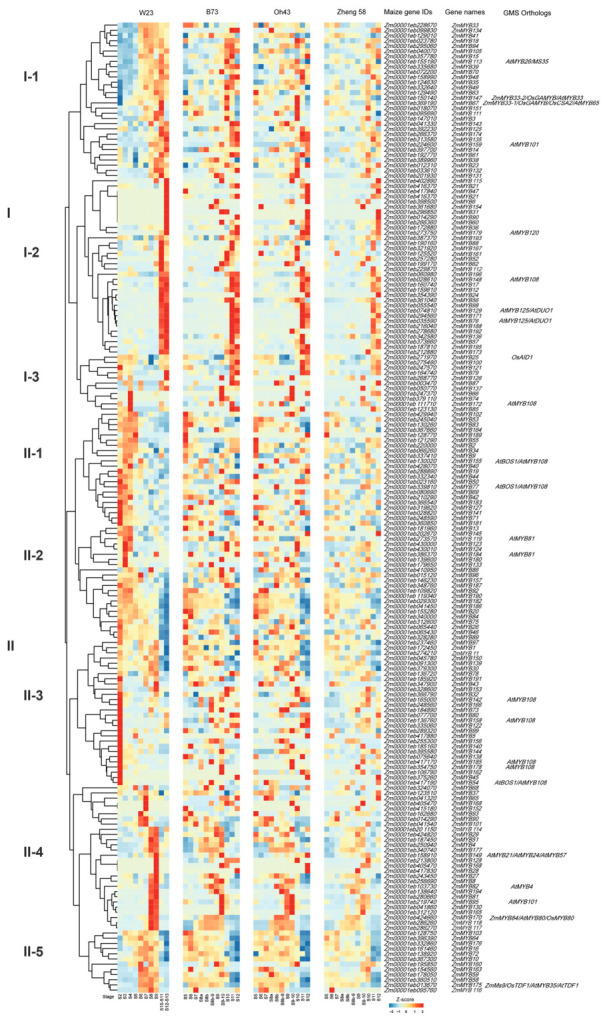
Expression analysis of MYB TFs in maize. Expression analysis of MYB TFs in maize based on RNA-seq data in maize inbred lines W23, B73, Oh43, and Zheng58. These genes are clustered into two clusters. Genes in cluster I and II were clustered into three and five sub-clusters, respectively.

Since the biological functions of genes are often associated with their spatiotemporal expression patterns, bioinformatic analyses provide useful information for investigating the functions of maize MYB TFs [19]. Transcriptomic analysis using RNA sequencing (RNA-seq) data is an effective approach for predicting the functions of unknown genes. To predict the functions of maize MYB TFs involved in anther and pollen development, we investigated the expression patterns of all maize MYB TFs during anther development using RNA-seq data from developing anthers of W23 (stages S2 to S12), B73, Oh43, and Zheng58 (stages S5 to S12) [19,22,69,70] (Figure 6).

Based on the RNA-seq data, all maize MYB TFs could be divided into two major clusters (Cluster I and Cluster II). Cluster I contained three subclusters (Subcluster I-1, I-2, and I-3). Subcluster I-1 comprised 29 MYB TFs that were expressed in the middle and late stages of maize anther development (starting from stage S6). Among them, including *ZmMYB147* (also designated as *ZmMYB33-2*) and *ZmMYB67* (also designated as *ZmMYB33-1*), *ZmMYB113*, *ZmMYB147*, *ZmMYB67*, and *ZmMYB159* are orthologous to the male sterility-related genes *AtMYB26/MS35*, *OsGAMYB*, *OsCSA2*, and *AtMYB101* [17,21,37,41,44,71,72]. Subcluster I-2 contained 34 MYB TFs whose expression peaks occurred during late anther development (stages S10 to S12). Among them, *ZmMYB179*, *ZmMYB148*, *ZmMYB129*, *ZmMYB76*, and *ZmMYB25* are orthologous to the male sterility-related genes *AtMYB120*, *AtMYB108*, *OsAID1*, and *AtMYB125/DUO1* [16,40,44,73], indicating that these genes may be essential for anther development. Notably, *ZmMYB31* and *ZmMYB90* are specifically upregulated at stage S12 of anther development, with negligible expression at other developmental stages. Subcluster I-3 contained 10 MYB TFs with expression peaks in both early (stages S2–S3) and late (stages S10–S12) anther development. *ZmMYB172* is orthologous to the male sterility-related gene *AtMYB108* [40], suggesting that it may also be essential for anther development (Figure 6).

Cluster II contained five subclusters (Subcluster II-1 to II-5). Subclusters II-1 and II-2 each contained 22 genes that were highly expressed during early anther development in W23 (stages S2–S5) but lowly expressed in Zheng58 anthers, indicating that the expression of some genes may be related to genetic background. Subcluster II-2 exhibited a higher expression level than Subcluster II-1 in the middle stage of anther development. *ZmMYB155*, *ZmMYB77*, *ZmMYB119*, and *ZmMYB184* are orthologous to the male sterility-related genes *AtMYB108/AtBOS1* and *AtMYB81* [15,40]. Subcluster II-3 contained 28 MYB TFs that displayed the highest expression among all clusters in early anther development (stage S2). Among them, *ZmMYB97* regulates pollen tube development, *ZmMYB150* is a male sterility-related gene, and *ZmMYB142*, *ZmMYB158*, *ZmMYB185*, *ZmMYB178*, and *ZmMYB54* are orthologous to the male sterility-related gene *AtMYB108/AtBOS1* [40]. Subcluster II-4 contained 30 genes that were highly expressed in the middle and late stages of anther development (stages S7–S10), although some of these genes showed expression peaks at specific stages in different inbred lines. For instance, *ZmMYB27* was highly expressed at stage S12 in B73 and Oh43 anthers but lowly expressed in Zheng58 anthers. Notably, three MYB TFs (*ZmMYB149*, *ZmMYB82*, *ZmMYB95*) are orthologous to the male sterility-related genes *AtMYB21/AtMYB24/AtMYB57*, *AtMYB101*, and *AtMYB4*, respectively, implying their potential involvement in anther development and male fertility regulation [32,38,39,40,44]. Subcluster II-5 contained 12 MYB TFs that were highly expressed in the middle stage of anther development (stages S5–S10), Among them, including *ZmMYB175* (also designated as *ZmMs9*), *ZmMYB175* is orthologous to the male sterility-related genes *AtMYB35*/*AtTDF1*, *OsTDF1* [18,23,24] (Figure 6). These results suggest that these MYB TFs may be essential for maize anther development and male fertility, although further experimental verification is required.

## 3. Discussion

Our phylogenetic tree, constructed from the domesticated maize (*Zea mays* ssp. *mays*) reference genome, should be interpreted in light of the evolutionary history of its wild progenitor, teosinte (*Zea mays* ssp. *parviglumis*). Maize was domesticated from teosinte approximately 9000 years ago, undergoing strong selective sweeps that dramatically altered plant and inflorescence architecture [74]. MYB TFs, which regulate diverse developmental processes including floral organ formation and pigmentation, may have been subject to similar selective pressures during domestication [10,11]. However, as our current analysis is confined to domesticated maize, future studies integrating teosinte genomic data will be essential to definitively assess the role of *MYB* gene family evolution in maize domestication.

MYB TFs play pivotal roles in diverse biological processes, including biotic and abiotic stress responses, plant signal transduction, and the biosynthesis of sporopollenin and protective waterproof barriers in various organs [1,10,11,49]. While functional studies of MYB TFs in *A. thaliana* and *O. sativa* have been relatively comprehensive and systematic, the functional characterization of maize MYB TFs remains largely unexplored. This study presents three major advances that distinguish it from previous work. First, building on the latest maize B73 reference genome, we constructed a high-confidence phylogenetic tree and comprehensive GO annotation for all 196 maize MYB TFs, establishing a solid foundation for future functional investigation. Second, we developed an integrated cross-species comparative workflow that systematically maps functionally characterized MYB TFs from *Arabidopsis* and rice onto their maize orthologs, and combined this with multi-inbred-line transcriptomic profiling of developing anthers to prioritize candidate genes likely involved in male reproductive development. This homology-guided and expression-based prediction strategy provides a paradigm for translating the rich functional knowledge accumulated in model plants to crops with less-characterized genomes. Third, we systematically summarized the diverse regulatory functions of MYB TFs across multiple biological pathways, including anther and pollen development, vegetative and seed development, stress responses, and secondary metabolism, providing a comprehensive reference resource for the plant MYB research community.

Among the candidate genes prioritized by our workflow, two are particularly noteworthy for their stage-specific expression patterns and evolutionary conservation with known male-fertility regulators in *Arabidopsis*. The first is *ZmMYB113* (*Zm00001eb155190*), the maize ortholog of *AtMYB26*-a transcription factor that regulates anther dehiscence through the transcriptional control of secondary wall thickening in the endothecium (Table S2). However, the function of *ZmMYB113* in maize anther development has not been previously reported. Our transcriptomic analysis across four independent maize anther datasets reveals that *ZmMYB113* exhibits a sharp expression peak specifically at the S10 stage-a critical phase corresponding to the onset of secondary wall thickening and pollen maturation. This highly reproducible spatiotemporal expression signature strongly suggests that *ZmMYB113* may play a conserved role in maize anther dehiscence, making it a prime candidate for future functional dissection of male sterility. The second is *ZmMYB159* (*Zm00001eb224600*), which shares clear orthology with AtMYB101-an *Arabidopsis* MYB implicated in pollen tube-synergid interaction during double fertilization (Table S2). Unlike *ZmMYB113*, which peaks at S10, *ZmMYB159* reaches its highest expression at late stages of anther development (post-S10), a temporal window that coincides with pollen maturation and the acquisition of pollination competence. This late-stage peak suggests that *ZmMYB159* may participate in the later phases of male reproductive development, potentially affecting pollen function rather than early anther morphogenesis. Together, these two genes exemplify how our comparative workflow can efficiently translate functional knowledge from model species to maize and generate testable hypotheses for experimental validation. Their distinct expression dynamics also highlight the functional diversification of MYB paralogs during maize anther development.

Looking forward, several research directions merit further investigation. The functional predictions generated by our bioinformatic pipeline, particularly those for Cluster I and Cluster II candidate genes orthologous to known male sterility regulators, await experimental validation through CRISPR/Cas9-mediated targeted mutagenesis or gene silencing in maize. Single-cell and spatial transcriptomics of developing anthers will be essential to resolve the precise cellular expression patterns and functional differentiation of MYB paralogs within specific anther cell types. Beyond cellular expression patterns, a key evolutionary question arising from our comparative analysis is whether maize and rice follow largely the same trends in MYB-mediated regulation of male fertility. Our orthologous mapping suggests that this is indeed the case, as exemplified by the conserved functional pairs *ZmMYB84/OsMYB80* and *ZmMYB33/OsGAMYB*. However, the extent to which their downstream target networks have diverged during evolution remains largely unexplored. A systematic comparison of the transcriptional regulons controlled by these MYB orthologs between maize and rice-through combined approaches such as DAP-seq, RNA-seq, and reciprocal complementation tests-would be a logical next step, which we are actively pursuing in our ongoing research. Such a comparative approach will not only clarify the degree of functional conservation versus divergence, but also inform the transferability of gene function knowledge from rice to maize.

Beyond comparative genomics, the regulatory mechanisms upstream of the predicted MYB candidates, including their interactions with phytohormone signaling pathways such as gibberellin, jasmonic acid, and brassinosteroid cascades, warrant systematic characterization. From an applied perspective, the validated male sterility-related MYB TFs hold promising potential as molecular tools for engineering male sterile lines in maize hybrid breeding programs, particularly when combined with conditional expression systems or environment-responsive promoters. Ultimately, the cross-species comparative workflow established in this study can be readily extended to the functional prediction and prioritization of other large transcription factor families in maize and beyond, accelerating the translation of model plant functional genomics into crop improvement.

## 4. Materials and Methods

### 4.1. Identification of MYB Transcription Factors in the Maize Genome

To comprehensively identify MYB transcription factors in the maize genome, we employed a rigorous double-identification strategy. First, a hidden Markov model (HMM) search was performed using the HMMER program (v3.3) with the MYB domain profile (PF00249) obtained from the Pfam database (https://pfam.xfam.org/) against the maize reference genome (RefGen_v5). Second, local BLASTP searches (E-value ≤ 10^−5^) NCBI BLAST+ (version 2.14.0) with characterized MYB amino acid sequences from *Arabidopsis* thaliana and *Oryza sativa* as queries. The resulting candidate sequences were merged and filtered to remove redundancies. To ensure the integrity of the conserved MYB domains, all candidates were further validated using the NCBI Conserved Domain Database (NCBI-CDD; https://www.ncbi.nlm.nih.gov/cdd, accessed on 2 March 2026) and SMART (http://smart.embl-heidelberg.de/, accessed on 2 March 2026). Only sequences containing at least one intact MYB DNA-binding domain were retained for subsequent analyses. In addition, previously annotated *MYB* gene names from the Gramene database (https://www.gramene.org/, accessed on 12 March 2026) and MaizeGDB (https://maizegdb.org/, accessed on 14 March 2026) were cross-referenced as a supplementary check to ensure complete coverage. The chromosomal locations of the identified *ZmMYB* genes were retrieved from the MaizeGDB genome browser based on the RefGen_v5 assembly (Zm-B73-REFERENCE-NAM-5.0).

### 4.2. Phylogenetic and GO Analysis of Maize MYB TFs

Phylogenetic trees were constructed with MEGA 11 (Version 11.0.13) using the maximum likelihood method, and bootstrap values were based on 500 replicates. The GO analysis was performed by GENE ONTOLOGY (http://geneontology.org/, accessed on 31 March 2026).

### 4.3. Transcriptome Data Processing and Differential Expression Analysis

The transcriptomic datasets used in this study were derived from previously published studies [19,22,69,70]. Total RNA was extracted from maize tissues using TRIzol reagent (Invitrogen, Carlsbad, CA, USA) following the manufacturer’s protocol. RNA quality, purity, and integrity were assessed using a NanoDrop 2000 spectrophotometer (Thermo Fisher Scientific, Waltham, MA, USA) and an Agilent 2100 Bioanalyzer (Agilent Technologies, Santa Clara, CA, USA); only samples with an RNA integrity number (RIN) ≥ 8.0 were used for subsequent analyses. Stranded RNA-seq libraries were constructed using the Illumina TruSeq RNA Library Prep Kit and sequenced on the Illumina NovaSeq 6000 platform, generating 150 bp paired-end reads. Raw reads were filtered using fastp (v0.23.2) to remove adapters, low-quality reads (Q < 20), and short reads. Clean reads were aligned to the maize reference genome (B73 RefGen_v5) using HISAT2 (v2.2.1) with default parameters. To profile spatial and temporal expression patterns, transcriptomic data from diverse tissues—including roots, stems, leaves, tassels, and staged anthers—were integrated from public datasets and our in-house sequencing. Gene expression levels were quantified as FPKM. Differentially expressed genes (DEGs) between tissues and developmental stages were identified using DESeq2 (v1.38.0), with thresholds of |log_2_FC| ≥ 1 and adjusted *p* < 0.05 (Benjamini–Hochberg).

## Data Availability

All data are shown in the main manuscript and in the Supplementary Materials.

## Supplementary Materials

The following supporting information can be downloaded at: https://www.mdpi.com/article/10.3390/ijms27135822/s1. References [39,68,75,76,77,78,79,80,81,82,83,84,85,86,87,88,89,90,91,92,93,94,95,96,97,98,99,100,101,102,103,104,105,106,107,108,109,110,111,112,113,114,115,116,117] are cited in the supplementary materials.
