# Supplementary material for: Genome-Wide Study of MYB Transcription Factors in Maize and Their Essential Roles in Male Fertility and Other Biological Processes
Source: Int J Mol Sci. 2026 Jun 27;27(13):5822. doi: 10.3390/ijms27135822 (PMC13360694; doi:10.3390/ijms27135822)
Supplement: Supplementary file 1 [file ijms-27-05822-s001.zip › Supplementary Figures-v3.pdf]

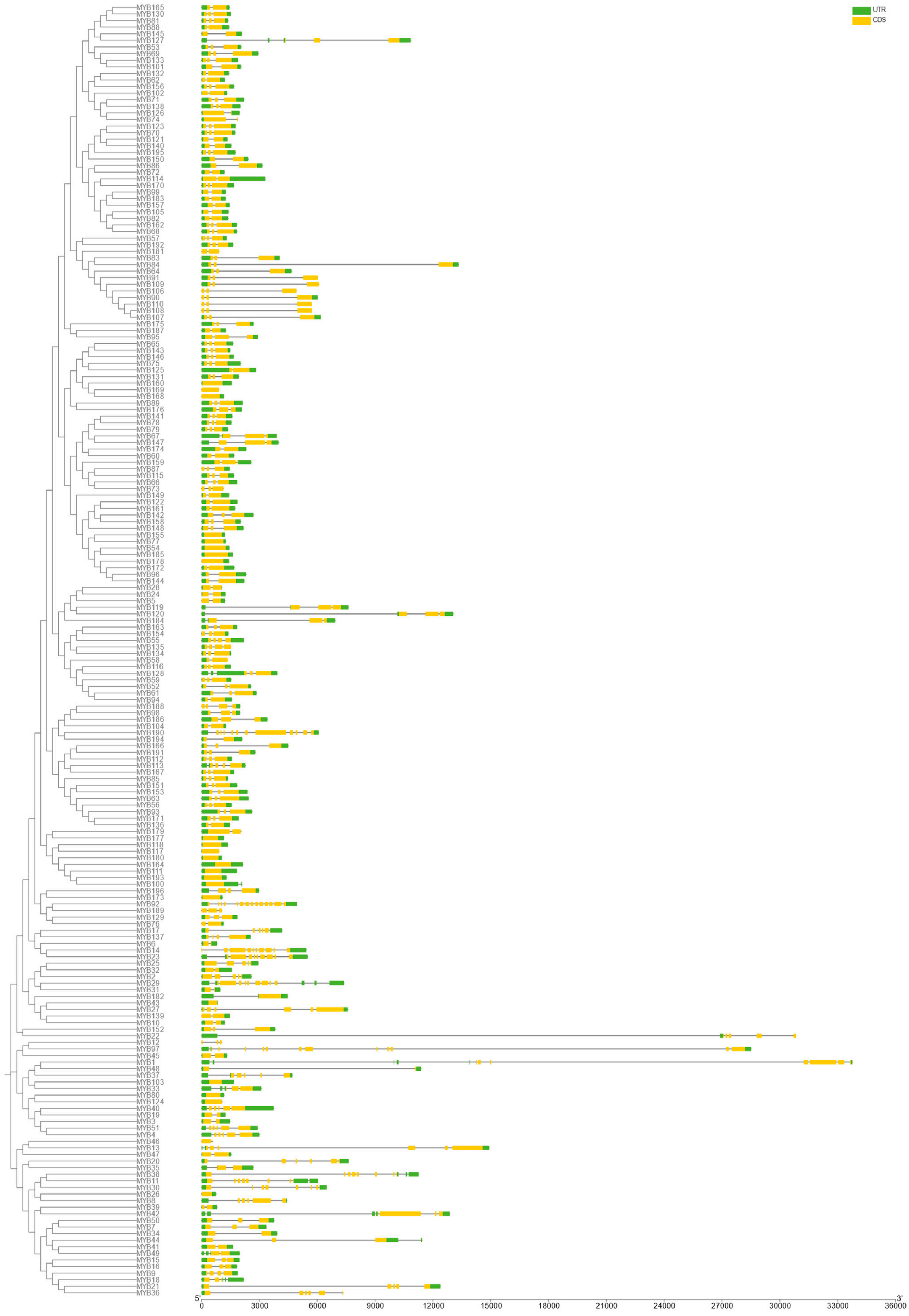

MYB165
MYB130
MYB81
MYB88
MYB145
MYB127
MYB53
MYB69
MYB133
MYB101
MYB132
MYB62
MYB156
MYB102
MYB71
MYB138
MYB126
MYB74
MYB123
MYB70
MYB121
MYB140
MYB195
MYB150
MYB86
MYB72
MYB114
MYB170
MYB99
MYB183
MYB157
MYB105
MYB82
MYB162
MYB68
MYB57
MYB192
MYB181
MYB83
MYB84
MYB64
MYB91
MYB109
MYB106
MYB90
MYB110
MYB108
MYB107
MYB175
MYB187
MYB95
MYB65
MYB143
MYB146
MYB75
MYB125
MYB131
MYB160
MYB169
MYB168
MYB89
MYB176
MYB141
MYB78
MYB79
MYB67
MYB147
MYB174
MYB60
MYB159
MYB87
MYB115
MYB66
MYB73
MYB149
MYB122
MYB161
MYB142
MYB158
MYB148
MYB155
MYB77
MYB54
MYB185
MYB178
MYB172
MYB96
MYB144
MYB28
MYB24
MYB5
MYB119
MYB120
MYB184
MYB163
MYB154
MYB55
MYB135
MYB134
MYB58
MYB116
MYB128
MYB59
MYB52
MYB61
MYB94
MYB188
MYB98
MYB186
MYB104
MYB190
MYB194
MYB166
MYB191
MYB112
MYB113
MYB167
MYB85
MYB151
MYB153
MYB63
MYB56
MYB93
MYB171
MYB136
MYB179
MYB177
MYB118
MYB117
MYB180
MYB164
MYB111
MYB193
MYB100
MYB196
MYB173
MYB92
MYB189
MYB129
MYB76
MYB17
MYB137
MYB6
MYB14
MYB23
MYB25
MYB32
MYB2
MYB29
MYB31
MYB182
MYB43
MYB27
MYB139
MYB10
MYB152
MYB22
MYB12
MYB97
MYB45
MYB1
MYB48
MYB37
MYB103
MYB33
MYB80
MYB124
MYB40
MYB19
MYB3
MYB51
MYB4
MYB46
MYB13
MYB47
MYB20
MYB35
MYB38
MYB11
MYB30
MYB26
MYB8
MYB39
MYB42
MYB50
MYB7
MYB34
MYB44
MYB41
MYB49
MYB15
MYB16
MYB9
MYB18
MYB21
MYB36
UTR
CDS
5'
3'
0
3000
6000
9000
12000
15000
18000
21000
24000
27000
30000
33000
36000

Figure S1. Structural features of exon-intron organizations in *Zea mays MYB* genes. Schematic representation of the genomic and transcript structures of *ZmMYB* gene family members. The lengths and positions of coding sequences (CDS) are indicated by green boxes, untranslated regions (UTRs) are denoted by blue boxes, and introns are represented by solid grey lines. The scale bar at the bottom indicates the length of the nucleotide sequences in base pairs (bp).

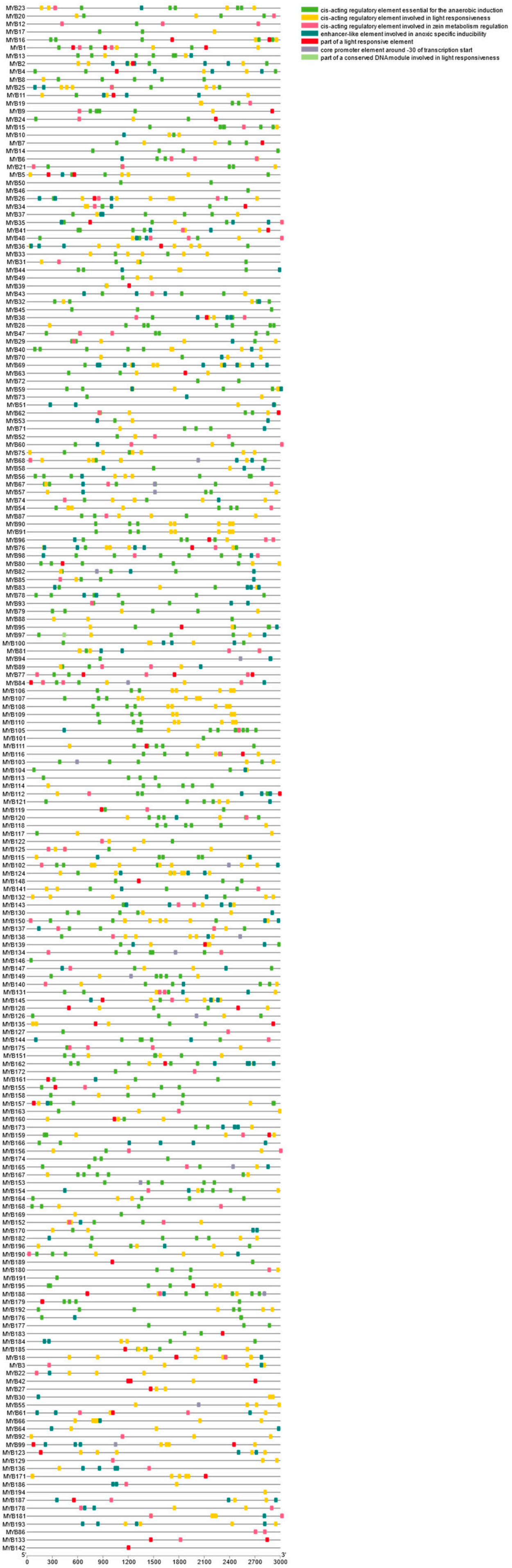
cis-acting regulatory element essential for the anaerobic induction
cis-acting regulatory element involved in light responsiveness
cis-acting regulatory element involved in zein metabolism regulation
enhancer-like element involved in anoxic specific inducibility
part of a light responsive element
core promoter element around -30 of transcription start
part of a conserved DNA module involved in light responsiveness
MYB23
MYB20
MYB12
MYB17
MYB16
MYB1
MYB13
MYB2
MYB4
MYB8
MYB25
MYB11
MYB19
MYB9
MYB24
MYB15
MYB10
MYB7
MYB14
MYB6
MYB21
MYB5
MYB50
MYB46
MYB26
MYB34
MYB37
MYB35
MYB41
MYB48
MYB36
MYB33
MYB31
MYB44
MYB49
MYB39
MYB43
MYB32
MYB45
MYB38
MYB28
MYB47
MYB29
MYB40
MYB70
MYB69
MYB63
MYB72
MYB59
MYB73
MYB51
MYB62
MYB53
MYB71
MYB52
MYB60
MYB75
MYB68
MYB58
MYB56
MYB67
MYB57
MYB74
MYB54
MYB87
MYB90
MYB91
MYB96
MYB76
MYB98
MYB80
MYB82
MYB85
MYB83
MYB78
MYB93
MYB79
MYB88
MYB95
MYB97
MYB100
MYB81
MYB94
MYB89
MYB77
MYB84
MYB106
MYB107
MYB108
MYB109
MYB110
MYB105
MYB101
MYB111
MYB116
MYB103
MYB104
MYB113
MYB114
MYB112
MYB121
MYB119
MYB120
MYB118
MYB117
MYB122
MYB125
MYB115
MYB102
MYB124
MYB148
MYB141
MYB132
MYB143
MYB130
MYB150
MYB137
MYB138
MYB139
MYB134
MYB146
MYB147
MYB149
MYB140
MYB131
MYB145
MYB128
MYB126
MYB135
MYB127
MYB144
MYB175
MYB151
MYB162
MYB172
MYB161
MYB155
MYB158
MYB157
MYB163
MYB160
MYB173
MYB159
MYB166
MYB156
MYB174
MYB165
MYB167
MYB153
MYB154
MYB164
MYB168
MYB169
MYB152
MYB170
MYB182
MYB196
MYB190
MYB189
MYB180
MYB191
MYB195
MYB188
MYB179
MYB192
MYB176
MYB177
MYB183
MYB184
MYB185
MYB18
MYB3
MYB22
MYB42
MYB27
MYB30
MYB55
MYB61
MYB66
MYB64
MYB92
MYB99
MYB123
MYB129
MYB136
MYB171
MYB186
MYB194
MYB187
MYB178
MYB181
MYB193
MYB86
MYB133
MYB142
5'
3'
0
300
600
900
1200
1500
1800
2100
2400
2700
3000

Figure S2. Distribution of major *cis*-acting regulatory elements in the promoter regions of *ZmMYB* genes. Identification of specialized *cis*-acting elements within the 3,000 bp upstream promoter regions from the transcription start sites (TSS) of *ZmMYB* genes. Distinct functional categories of regulatory elements—including light responsiveness, anaerobic induction, hormone signaling, and stress defense—are color-coded as indicated in the right-hand legend. The scale indicates the distance upstream from the TSS.

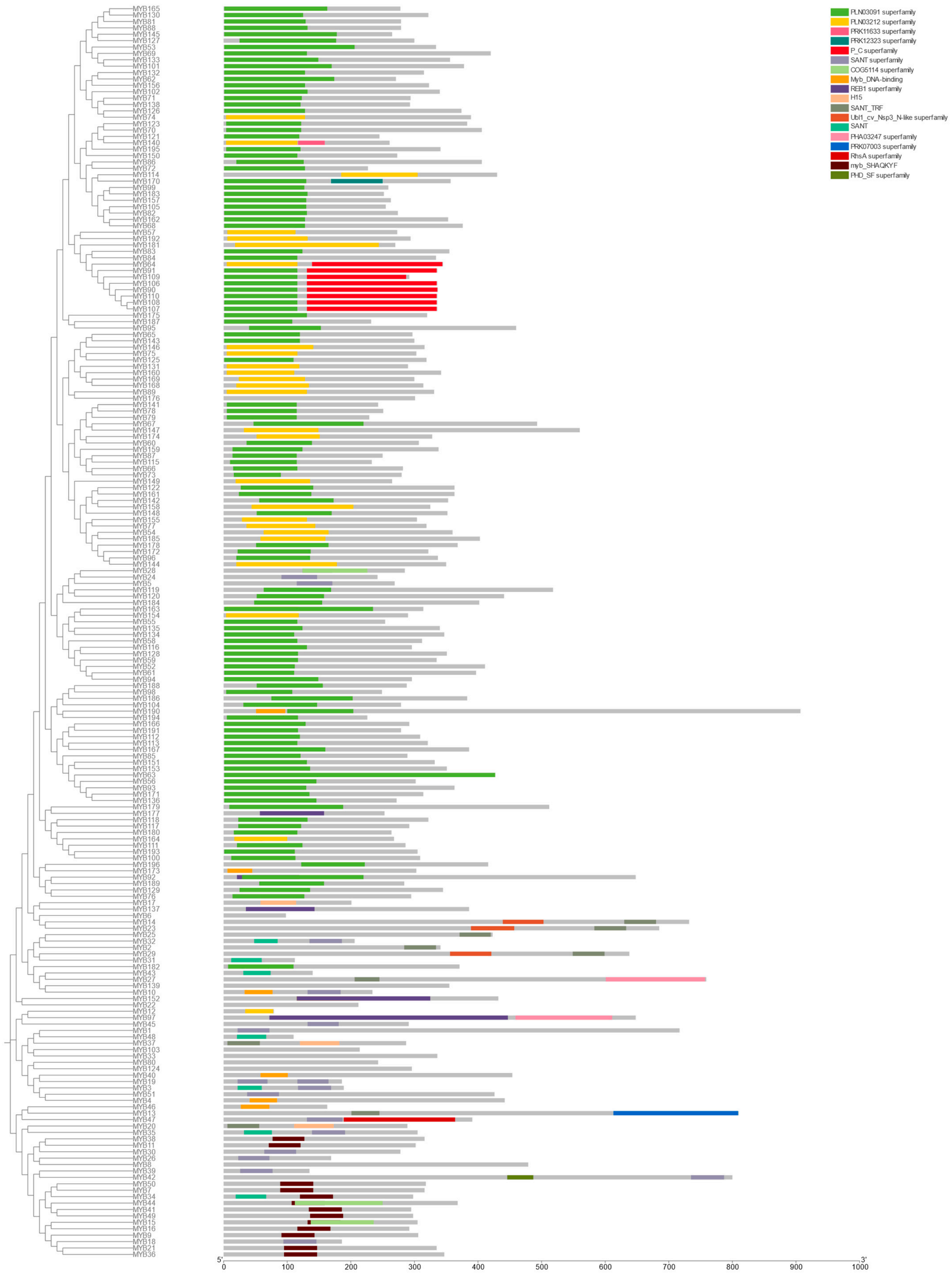

MYB165
MYB130
MYB81
MYB88
MYB145
MYB127
MYB53
MYB69
MYB133
MYB101
MYB132
MYB62
MYB156
MYB102
MYB71
MYB138
MYB126
MYB74
MYB123
MYB70
MYB121
MYB140
MYB195
MYB150
MYB86
MYB72
MYB114
MYB170
MYB99
MYB183
MYB157
MYB105
MYB82
MYB162
MYB68
MYB57
MYB192
MYB181
MYB83
MYB84
MYB64
MYB91
MYB109
MYB106
MYB90
MYB110
MYB108
MYB107
MYB175
MYB187
MYB95
MYB65
MYB143
MYB146
MYB75
MYB125
MYB131
MYB160
MYB169
MYB168
MYB89
MYB176
MYB141
MYB78
MYB79
MYB67
MYB147
MYB174
MYB60
MYB159
MYB87
MYB115
MYB66
MYB73
MYB149
MYB122
MYB161
MYB142
MYB158
MYB148
MYB155
MYB77
MYB54
MYB185
MYB178
MYB172
MYB96
MYB144
MYB28
MYB24
MYB5
MYB119
MYB120
MYB184
MYB163
MYB154
MYB55
MYB135
MYB134
MYB58
MYB116
MYB128
MYB59
MYB52
MYB61
MYB94
MYB188
MYB98
MYB186
MYB104
MYB190
MYB194
MYB166
MYB191
MYB112
MYB113
MYB167
MYB85
MYB151
MYB153
MYB63
MYB56
MYB93
MYB171
MYB136
MYB179
MYB177
MYB118
MYB117
MYB180
MYB164
MYB111
MYB193
MYB100
MYB196
MYB173
MYB92
MYB189
MYB129
MYB76
MYB17
MYB137
MYB6
MYB14
MYB23
MYB25
MYB32
MYB2
MYB29
MYB31
MYB182
MYB43
MYB27
MYB139
MYB10
MYB152
MYB22
MYB12
MYB97
MYB45
MYB1
MYB48
MYB37
MYB103
MYB33
MYB80
MYB124
MYB40
MYB19
MYB3
MYB51
MYB4
MYB46
MYB13
MYB47
MYB20
MYB35
MYB38
MYB11
MYB30
MYB26
MYB8
MYB39
MYB42
MYB50
MYB7
MYB34
MYB44
MYB41
MYB49
MYB15
MYB16
MYB9
MYB18
MYB21
MYB36
5'
0
100
200
300
400
500
600
700
800
900
1000
3'
PLN03091 superfamily
PLN03212 superfamily
PRK11633 superfamily
PRK12323 superfamily
P_C superfamily
SANT superfamily
COG5114 superfamily
Myb_DNA-binding
REB1 superfamily
H15
SANT_TRF
Ubl1_cv_Nsp3_N-like superfamily
SANT
PHA03247 superfamily
PRK07003 superfamily
RhsA superfamily
myb_SHAQKYF
PHD_SF superfamily

Figure S3. Alignment and conserved domain characterization of the ZmMYB proteins. Conserved Domain Database (CDD) and motif analysis of the ZmMYB transcription factor family. The architectural distribution reveals highly conserved MYB DNA-binding domains across the protein sequences, highlighting the evolutionary preservation of key functional modules essential for DNA binding and transcriptional regulation.
