# Supplementary material for: Genome-Wide Study of MYB Transcription Factors in Maize and Their Essential Roles in Male Fertility and Other Biological Processes
Source: Int J Mol Sci. 2026 Jun 27;27(13):5822. doi: 10.3390/ijms27135822 (PMC13360694; doi:10.3390/ijms27135822)
Supplement: Supplementary file 1 [file ijms-27-05822-s001.zip › Supplementary Table S1 The maize MYB family MYB genes based on B73 reference genome V5.0 and expression patterns-v1.pdf]

Table S1. The maize *MYB* family: *MYB* genes based on B73 reference genome V5.0 and expression patterns.

No.	Name	Gene ID (B73 V5)[1]	Chromosomal localization		Expression Patterns[2]											
			chr	pos	Root	Leaf	Stem and Sam	Internode	Tassel	Anther	Silk	Cob	Seed	Endosperm	Embryo	Pericarp
1	*ZmMYB1*	*Zm00001eb172450*	4	34451947	+	+	+	+	-	+	+	+	+	-	+	+
2	*ZmMYB2*	*Zm00001eb220000*	5	21962347	+	+	+	+	+	+	+	+	+	+	+	+
3	*ZmMYB3*	*Zm00001eb147010*	3	181227276	+	+	-	+	+	-	+	-	+	+	+	+
4	*ZmMYB4*	*Zm00001eb250940*	5	205536128	+	+	-	+	+	+	+	+	+	-	+	+
5	*ZmMYB5*	*Zm00001eb417880*	10	90925352	-	-	-	-	-	-	-	-	+	+	+	+
6	*ZmMYB6*	*Zm00001eb398500*	9	148342953	+	+	+	-	-	-	-	-	-	-	-	-
7	*ZmMYB7*	*Zm00001eb391130*	9	120693131												
8	*ZmMYB8*	*Zm00001eb256690*	5	220063974	+	+	+	+	+	+	+	+	+	+	+	+
9	*ZmMYB9*	*Zm00001eb337410*	8	21157918	+	+	+	+	+	+	+	+	+	-	+	+
10	*ZmMYB10*	*Zm00001eb367300*	8	173983699	+	+	+	+	+	+	+	+	+	-	+	+
11	*ZmMYB11*	*Zm00001eb274210*	6	102804719	+	+	+	+	+	+	+	+	+	+	+	+
12	*ZmMYB12*	*Zm00001eb159610*	3	223547340	+	+	+	+	+	+	+	+	+	-	-	+
13	*ZmMYB13*	*Zm00001eb181960*	4	120023470	+	+	+	+	+	+	+	+	+	+	+	+
14	*ZmMYB14*	*Zm00001eb397700*	9	145818645	+	+	+	+	+	+	+	+	+	+	+	+
15	*ZmMYB15*	*Zm00001eb357780*	8	145619088	+	+	+	+	+	+	+	+	+	+	-	+
16	*ZmMYB16*	*Zm00001eb161460*	3	229301492	+	+	+	+	+	+	+	+	+	+	+	+
17	*ZmMYB17*	*Zm00001eb160740*	3	227194759	+	+	+	+	+	+	+	+	+	+	+	+
18	*ZmMYB18*	*Zm00001eb023780*	1	95697323												
19	*ZmMYB19*	*Zm00001eb288890*	6	160476634	+	-	-	-	-	-	-	-	+	+	+	-
20	*ZmMYB20*	*Zm00001eb155280*	3	209669718	+	+	+	+	+	+	+	+	+	+	+	+
21	*ZmMYB21*	*Zm00001eb416370*	10	82869996												
22	*ZmMYB22*	*Zm00001eb416380*	10	82907347	+	+	+	+	+	+	+	+	+	+	+	+
23	*ZmMYB23*	*Zm00001eb012310*	1	40290648	+	+	+	+	+	+	+	+	+	+	+	+
24	*ZmMYB24*	*Zm00001eb354390*	8	132005876	-	-	-	-	-	-	-	-	+	+	+	+
25	*ZmMYB25*	*Zm00001eb271970*	6	92123668	+	+	+	+	+	+	+	+	+	+	+	+
26	*ZmMYB26*	*Zm00001eb065440*	1	307828915	+	+	-	+	-	-	-	-	-	-	-	-
27	*ZmMYB27*	*Zm00001eb243450*	5	175086894	+	+	+	+	+	+	+	+	+	+	+	+
28	*ZmMYB28*	*Zm00001eb417830*	10	90503707	-	-	-	-	-	-	-	-	+	+	-	+
29	*ZmMYB29*	*Zm00001eb424820*	10	127598663	+	+	+	+	+	+	+	+	+	+	+	+
30	*ZmMYB30*	*Zm00001eb379300*	9	29215205	+	+	+	+	+	+	+	+	+	+	+	+

Table S1. The maize *MYB* family: *MYB* genes based on B73 reference genome V5.0 and expression patterns.																
No.	Name	Gene ID (B73 V5)[1]	Chromosomal localization		Expression Patterns[2]											
			chr	pos	Root	Leaf	Stem and Sam	Internode	Tassel	Anther	Silk	Cob	Seed	Endosperm	Embryo	Pericarp
31	*ZmMYB31*	*Zm00001eb296850*	6	177712798	-	+	+	-	-	-	-	-	-	-	-	-
32	*ZmMYB32*	*Zm00001eb366790*	8	173153442	+	+	-	+	+	+	-	+	+	+	+	+
33	*ZmMYB33*	*Zm00001eb228670*	5	66039016	+	+	+	+	+	+	+	+	+	+	+	+
34	*ZmMYB34*	*Zm00001eb066260*	2	1946187	+	+	+	+	+	+	+	+	+	+	+	+
35	*ZmMYB35*	*Zm00001eb124630*	3	19023966	+	+	+	+	+	+	+	+	+	+	+	+
36	*ZmMYB36*	*Zm00001eb172880*	4	36423414	+	+	+	+	+	+	+	+	+	+	+	+
37	*ZmMYB37*	*Zm00001eb123510*	3	14444415	+	+	+	+	+	+	+	+	+	+	+	+
38	*ZmMYB38*	*Zm00001eb389960*	9	115594109	+	+	+	+	+	+	+	+	+	+	+	+
39	*ZmMYB39*	*Zm00001eb335680*	8	14193858	+	+	+	+	+	+	+	-	+	-	-	-
40	*ZmMYB40*	*Zm00001eb428070*	10	137454470	+	+	+	+	+	+	+	+	+	+	+	+
41	*ZmMYB41*	*Zm00001eb129010*	3	45238602	+	+	+	+	+	+	+	+	+	+	-	-
42	*ZmMYB42*	*Zm00001eb210290*	5	514400	+	+	+	+	+	+	+	+	+	+	+	+
43	*ZmMYB43*	*Zm00001eb347900*	8	98800011	-	-	-	-	-	-	-	-	-	-	-	-
44	*ZmMYB44*	*Zm00001eb332340*	8	1130567	+	+	+	+	+	+	+	+	+	+	+	-
45	*ZmMYB45*	*Zm00001eb375260*	9	16662214	-	-	-	-	-	-	-	-	+	+	+	+
46	*ZmMYB46*	*Zm00001eb065430*	1	307773133	+	+	-	-	-	-	-	-	-	-	-	-
47	*ZmMYB47*	*Zm00001eb417840*	10	90538196	-	-	-	-	-	-	-	-	+	+	-	+
48	*ZmMYB48*	*Zm00001eb158990*	3	221713437	+	-	-	-	-	-	-	-	-	-	-	-
49	*ZmMYB49*	*Zm00001eb332640*	8	2146652	+	+	-	+	+	-	-	-	-	-	-	-
50	*ZmMYB50*	*Zm00001eb023160*	1	90579804	+	+	+	+	+	+	+	+	+	+	+	+
51	*ZmMYB51*	*Zm00001eb187450*	4	161834006	+	+	+	+	+	+	+	+	+	+	+	+
52	*ZmMYB52*	*Zm00001eb257280*	5	220969206	+	-	-	-	-	-	-	-	-	-	-	-
53	*ZmMYB53*	*Zm00001eb245040*	5	180300047	-	+	+	-	+	-	+	+	+	-	-	-
54	*ZmMYB54*	*Zm00001eb417190*	10	87120080	+	+	-	+	-	-	-	-	-	-	+	+
55	*ZmMYB55*	*Zm00001eb121290*	3	6674549	+	+	+	+	+	+	-	-	+	-	-	+
56	*ZmMYB56*	*Zm00001eb361040*	8	157455928	+	+	+	+	+	+	-	-	+	-	-	+
57	*ZmMYB57*	*Zm00001eb373660*	9	11117066	-	+	-	-	-	+	-	-	-	-	-	-
58	*ZmMYB58*	*Zm00001eb360510*	8	155283445	+	+	+	+	+	-	-	+	-	-	+	-
59	*ZmMYB59*	*Zm00001eb178050*	4	71737601	+	+	-	-	+	-	-	-	-	-	-	-
60	*ZmMYB60*	*Zm00001eb266360*	6	42655721	+	+	+	+	-	+	-	-	-	-	-	-

Table S1. The maize *MYB* family: *MYB* genes based on B73 reference genome V5.0 and expression patterns.

No.	Name	Gene ID (B73 V5)[1]	Chromosomal localization		Expression Patterns[2]											
			chr	pos	Root	Leaf	Stem and Sam	Internode	Tassel	Anther	Silk	Cob	Seed	Endosperm	Embryo	Pericarp
61	*ZmMYB61*	*Zm00001eb192770*	4	180400346	+	-	-	-	-	-	-	-	+	-	-	+
62	*ZmMYB62*	*Zm00001eb199170*	4	203243104	+	+	-	+	+	-	-	-	+	-	-	-
63	*ZmMYB63*	*Zm00001eb129490*	3	48922348	+	+	+	+	+	+	+	+	+	-	-	+
64	*ZmMYB64*	*Zm00001eb396390*	9	142267452	-	+	-	+	+	-	+	+	+	-	-	-
65	*ZmMYB65*	*Zm00001eb041320*	1	217666913	-	-	-	-	-	-	-	-	+	+	-	+
66	*ZmMYB66*	*Zm00001eb247370*	5	189181793	+	+	-	+	-	-	-	-	-	-	-	-
67	*ZmMYB67*	*Zm00001eb369190*	8	177675625	+	+	+	+	+	+	+	+	+	+	+	+
68	*ZmMYB68*	*Zm00001eb324070*	7	166182515	+	+	-	+	-	+	-	-	-	-	-	-
69	*ZmMYB69*	*Zm00001eb080690*	2	43568184	-	+	+	+	+	-	+	+	+	+	-	+
70	*ZmMYB70*	*Zm00001eb072200*	2	13838408	+	+	+	+	+	-	-	-	+	-	-	-
71	*ZmMYB71*	*Zm00001eb248590*	5	193010373	-	-	+	+	+	-	-	+	+	-	+	-
72	*ZmMYB72*	*Zm00001eb138920*	3	141758915	+	+	+	+	+	-	+	+	+	+	+	+
73	*ZmMYB73*	*Zm00001eb184890*	4	145998532	+	-	-	-	-	-	-	-	-	-	-	-
74	*ZmMYB74*	*Zm00001eb379110*	9	28652737	+	+	-	-	+	-	-	-	-	-	-	-
75	*ZmMYB75*	*Zm00001eb312600*	7	117111316	+	+	+	+	+	+	+	+	+	-	+	+
76	*ZmMYB76*	*Zm00001eb035590*	1	194469822	-	-	-	-	-	-	-	-	-	-	-	-
77	*ZmMYB77*	*Zm00001eb339810*	8	34045580	+	+	-	+	-	-	-	-	-	-	-	-
78	*ZmMYB78*	*Zm00001eb136720*	3	128792139	+	+	+	+	+	+	+	+	+	+	+	+
79	*ZmMYB79*	*Zm00001eb164740*	4	1837797	+	+	+	+	-	+	-	-	-	-	-	-
80	*ZmMYB80*	*Zm00001eb077700*	2	31057680	+	+	+	+	+	+	-	-	+	-	+	+
81	*ZmMYB81*	*Zm00001eb280660*	6	128839472	-	+	-	+	+	-	-	-	+	+	+	+
82	*ZmMYB82*	*Zm00001eb103730*	2	202730800	+	+	+	+	+	+	+	+	+	+	+	+
83	*ZmMYB83*	*Zm00001eb130260*	3	54961178	+	+	+	+	+	+	+	+	+	+	+	+
84	*ZmMYB84*	*Zm00001eb340000*	8	35604977	+	+	+	+	+	+	+	+	+	+	+	+
85	*ZmMYB85*	*Zm00001eb123130*	3	13176780	+	-	-	-	-	-	-	-	-	-	-	-
86	*ZmMYB86*	*Zm00001eb410950*	10	29058975	+	+	-	+	+	-	+	+	+	-	-	-
87	*ZmMYB87*	*Zm00001eb003470*	1	9192133	+	-	-	+	-	-	-	-	-	-	-	-
88	*ZmMYB88*	*Zm00001eb190160*	4	171999050	+	+	+	+	+	+	+	+	+	-	+	-
89	*ZmMYB89*	*Zm00001eb328280*	7	176663838	-	+	+	+	+	+	+	+	+	+	+	+
90	*ZmMYB90*	*Zm00001eb014290*	1	47948490	-	-	-	-	+	-	+	+	+	-	+	-

Table S1. The maize *MYB* family: *MYB* genes based on B73 reference genome V5.0 and expression patterns.																
No.	Name	Gene ID (B73 V5)[1]	Chromosomal localization		Expression Patterns[2]											
			chr	pos	Root	Leaf	Stem and Sam	Internode	Tassel	Anther	Silk	Cob	Seed	Endosperm	Embryo	Pericarp
91	*ZmMYB91*	*Zm00001eb014370*	1	47986957	-	-	-	-	+	-	+	+	+	-	+	-
92	*ZmMYB92*	*Zm00001eb109820*	2	219427428	+	+	+	+	+	+	+	+	+	+	+	+
93	*ZmMYB93*	*Zm00001eb162680*	3	233179317	+	+	+	+	+	-	+	+	+	-	-	-
94	*ZmMYB94*	*Zm00001eb295060*	6	174531169	+	+	+	+	+	+	-	+	+	-	+	+
95	*ZmMYB95*	*Zm00001eb219740*	5	21129197	+	-	-	+	+	-	-	-	+	+	+	+
96	*ZmMYB96*	*Zm00001eb015120*	1	51297898	+	+	+	+	-	-	-	-	-	-	-	-
97	*ZmMYB97*	*Zm00001eb237460*	5	137986868	+	+	+	+	+	+	+	+	+	+	+	+
98	*ZmMYB98*	*Zm00001eb055540*	1	276169671	+	+	+	+	+	+	-	-	+	-	-	+
99	*ZmMYB99*	*Zm00001eb289320*	6	161849746	+	+	+	+	+	-	+	-	+	+	-	-
100	*ZmMYB100*	*Zm00001eb275490*	6	107207628	+	+	+	+	+	+	+	+	+	+	+	+
101	*ZmMYB101*	*Zm00001eb041540*	1	218782726	-	+	+	+	+	-	+	+	+	-	-	-
102	*ZmMYB102*	*Zm00001eb429940*	10	142214302	+	-	-	-	-	-	-	-	-	-	-	-
103	*ZmMYB103*	*Zm00001eb128750*	3	43312685	+	-	+	+	-	-	-	-	-	-	+	-
104	*ZmMYB104*	*Zm00001eb128760*	3	43312858	+	-	+	+	-	-	-	-	-	-	+	-
105	*ZmMYB105*	*Zm00001eb040070*	1	211534896	+	+	+	-	-	+	-	-	-	-	-	-
106	*ZmMYB106*	*Zm00001eb014260*	1	47923929	-	-	-	-	+	-	+	-	+	-	-	-
107	*ZmMYB107*	*Zm00001eb014280*	1	47935437												
108	*ZmMYB108*	*Zm00001eb014320*	1	47961449												
109	*ZmMYB109*	*Zm00001eb014350*	1	47974272												
110	*ZmMYB110*	*Zm00001eb014390*	1	47999589	-	-	-	-	+	-	+	+	+	-	+	-
111	*ZmMYB111*	*Zm00001eb095690*	2	168274815	+	+	+	+	+	+	+	+	+	+	+	+
112	*ZmMYB112*	*Zm00001eb229870*	5	71295333	+	+	-	-	-	-	+	-	-	-	-	-
113	*ZmMYB113*	*Zm00001eb155190*	3	209458510	-	+	-	+	+	-	-	+	+	+	+	+
114	*ZmMYB114*	*Zm00001eb201150*	4	213191112	+	+	+	+	+	+	+	+	+	-	+	+
115	*ZmMYB115*	*Zm00001eb402890*	9	158540780	+	-	-	-	-	-	-		-	-	+	-
116	*ZmMYB116*	*Zm00001eb095760*	2	168711390	-	+	+	-	+	-	-	+	+	-	-	-
117	*ZmMYB117*	*Zm00001eb286270*	6	151816536	-	-	-	-	+	-	-	-	-	-	-	-
118	*ZmMYB118*	*Zm00001eb286260*	6	151785088	-	-	-	-	+	-	-	-	-	-	-	-
119	*ZmMYB119*	*Zm00001eb273570*	6	98848289	+	+	+	+	+	+	+	+	+	+	+	+
120	*ZmMYB120*	*Zm00001eb273580*	6	98951685	+	+	+	+	+	+	+	+	+	+	+	+

Table S1. The maize *MYB* family: *MYB* genes based on B73 reference genome V5.0 and expression patterns.

No.	Name	Gene ID (B73 V5)[1]	Chromosomal localization		Expression Patterns[2]											
			chr	pos	Root	Leaf	Stem and Sam	Internode	Tassel	Anther	Silk	Cob	Seed	Endosperm	Embryo	Pericarp
121	*ZmMYB121*	*Zm00001eb247570*	5	190173305	+	+	-	+	-	-	-	-	-	-	-	-
122	*ZmMYB122*	*Zm00001eb335060*	8	11932475	+	+	+	+	-	+	-	-	+	-	+	+
123	*ZmMYB123*	*Zm00001eb430000*	10	142325860	+	+	-	+	-	-	-	-	-	-	-	+
124	*ZmMYB124*	*Zm00001eb430010*	10	142326261	+	+	-	+	-	-	-	-	-	-	-	+
125	*ZmMYB125*	*Zm00001eb392230*	9	124772082	+	+	+	+	+	+	+	+	+	+	+	+
126	*ZmMYB126*	*Zm00001eb268770*	6	66819689	+	+	-	-	+	+	-	-	+	-	-	-
127	*ZmMYB127*	*Zm00001eb319620*	7	149364659	+	+	+	+	+	+	+	+	+	+	+	+
128	*ZmMYB128*	*Zm00001eb213800*	5	5854700	+	+	+	+	+	+	+	-	-	-	+	-
129	*ZmMYB129*	*Zm00001eb074810*	2	21560394	-	-	-	-	-	+	-	-	-	-	-	-
130	*ZmMYB130*	*Zm00001eb041860*	1	220215475	-	+	-	-	+	-	-	-	-	-	-	-
131	*ZmMYB131*	*Zm00001eb201930*	4	217615806	-	+	-	-	+	-	-	-	-	-	-	-
132	*ZmMYB132*	*Zm00001eb033610*	1	186611059	+	+	-	-	+	-	-	-	-	-	-	-
133	*ZmMYB133*	*Zm00001eb179650*	4	86106705	-	+	+	+	+	-	+	+	+	-	-	+
134	*ZmMYB134*	*Zm00001eb099830*	2	187758522	+	-	-	+	+	-	-	-	-	-	-	-
135	*ZmMYB135*	*Zm00001eb313580*	7	124076015	+	+	-	+	+	+	-	-	-	-	-	-
136	*ZmMYB136*	*Zm00001eb342580*	8	64627703	+	+	+	+	+	-	-	-	+	-	+	+
137	*ZmMYB137*	*Zm00001eb050770*	1	258509093	-	-	-	-	-	-	-	-	+	+	-	-
138	*ZmMYB138*	*Zm00001eb075640*	2	24441782	-	+	-	+	+	+	+	+	-	-	+	+
139	*ZmMYB139*	*Zm00001eb091300*	2	135410126	+	+	+	+	+	+	+	+	+	-	+	+
140	*ZmMYB140*	*Zm00001eb185160*	4	147804262	+	+	-	-	-	-	-	-	-	-	-	-
141	*ZmMYB141*	*Zm00001eb028820*	1	154476509	+	+	+	+	+	+	+	+	+	+	+	+
142	*ZmMYB142*	*Zm00001eb165000*	4	2905504	+	+	+	+	-	+	+	-	-	-	-	+
143	*ZmMYB143*	*Zm00001eb041330*	1	217727302	-	+	-	+	+	-	-	-	+	-	-	-
144	*ZmMYB144*	*Zm00001eb395580*	9	139733687	+	+	-	+	-	-	-	-	-	-	-	+
145	*ZmMYB145*	*Zm00001eb202670*	4	223202328	+	+	+	+	+	-	-	-	+	-	-	-
146	*ZmMYB146*	*Zm00001eb109860*	2	219520230												
147	*ZmMYB147*	*Zm00001eb150140*	3	192102234	+	+	-	+	+	+	+	+	+	+	+	+
148	*ZmMYB148*	*Zm00001eb028610*	1	152367682	-	+	-	-	+	+	+	+	+	+	-	+
149	*ZmMYB149*	*Zm00001eb158910*	3	221365129	-	-	-	-	-	+	+	+	+	-	-	-
150	*ZmMYB150*	*Zm00001eb045780*	1	236963527	+	+	+	+	+	-	-	+	-	-	+	-

Table S1. The maize *MYB* family: *MYB* genes based on B73 reference genome V5.0 and expression patterns.																
No.	Name	Gene ID (B73 V5)[1]	Chromosomal localization		Expression Patterns[2]											
			chr	pos	Root	Leaf	Stem and Sam	Internode	Tassel	Anther	Silk	Cob	Seed	Endosperm	Embryo	Pericarp
151	*ZmMYB151*	*Zm00001eb018070*	1	64371137	-	+	+	+	+	-	+	-	+	-	-	-
152	*ZmMYB152*	*Zm00001eb415180*	10	75717760	-	-	-	-	-	-	-	-	+	+	-	+
153	*ZmMYB153*	*Zm00001eb328600*	7	177348968	+	+	+	+	+	+	+	+	+	-	-	+
154	*ZmMYB154*	*Zm00001eb361680*	8	159670916	+	-	-	-	-	-	-	-	-	-	-	-
155	*ZmMYB155*	*Zm00001eb130020*	3	52802108	+	+	-	+	-	-	-	-	-	-	-	+
156	*ZmMYB156*	*Zm00001eb255300*	5	216685538	+	+	-	-	+	-	-	-	+	-	-	-
157	*ZmMYB157*	*Zm00001eb146230*	3	178217240	+	+	+	+	+	+	+	-	+	-	+	+
158	*ZmMYB158*	*Zm00001eb136760*	3	129136771	-	+	-	-	-	+	+	-	-	-	-	+
159	*ZmMYB159*	*Zm00001eb224600*	5	43154988	-	-	-	-	-	+	-	-	-	-	-	-
160	*ZmMYB160*	*Zm00001eb195850*	4	191051305	-	+	-	-	-	-	-	-	-	-	-	+
161	*ZmMYB161*	*Zm00001eb125520*	3	23687239	+	+	+	+	+	-	+	+	+	-	-	+
162	*ZmMYB162*	*Zm00001eb106790*	2	211638635	+	-	-	+	-	-	-	-	-	-	-	-
163	*ZmMYB163*	*Zm00001eb154560*	3	207197815	+	+	-	-	-	-	-	-	-	-	-	-
164	*ZmMYB164*	*Zm00001eb367660*	8	174521739	+	+	+	+	+	+	+	+	+	+	+	+
165	*ZmMYB165*	*Zm00001eb312120*	7	113206300	+	-	-	-	+	-	-	-	-	-	-	-
166	*ZmMYB166*	*Zm00001eb248560*	5	192959147	+	+	+	+	+	+	-	-	+	+	+	+
167	*ZmMYB167*	*Zm00001eb321920*	7	156552270	+	-	-	-	-	-	-	-	+	+	-	-
168	*ZmMYB168*	*Zm00001eb405470*	10	2171060	-	-	-	-	-	-	-	-	-	-	-	-
169	*ZmMYB169*	*Zm00001eb405510*	10	2177185	-	-	-	-	-	-	-	-	-	-	-	-
170	*ZmMYB170*	*Zm00001eb424660*	10	126882091	+	-	-	+	+	-	-	-	-	-	+	+
171	*ZmMYB171*	*Zm00001eb294560*	6	173614872	+	+	+	+	-	+	-	-	+	-	-	+
172	*ZmMYB172*	*Zm00001eb111710*	2	224221614	+	+	-	+	-	-	-	-	-	-	-	-
173	*ZmMYB173*	*Zm00001eb212880*	5	4444783	+	-	-	-	-	-	-	-	-	-	-	-
174	*ZmMYB174*	*Zm00001eb266370*	6	42757910	+	+	+	+	-	-	-	-	-	-	-	-
175	*ZmMYB175*	*Zm00001eb013670*	1	45775586	-	-	-	-	+	-	-	-	+	-	+	-
176	*ZmMYB176*	*Zm00001eb332860*	8	3092516	+	+	+	+	+	+	+	+	+	+	+	-
177	*ZmMYB177*	*Zm00001eb340740*	8	41647480	-	-	-	-	+	-	-	-	-	-	-	-
178	*ZmMYB178*	*Zm00001eb354750*	8	133732393	-	-	-	-	-	-	-	-	-	-	-	-
179	*ZmMYB179*	*Zm00001eb273750*	6	100578377	-	-	-	-	-	+	-	-	-	-	-	-
180	*ZmMYB180*	*Zm00001eb139600*	3	145207627	-	-	-	-	-	-	-	-	+	+	-	-

Table S1. The maize *MYB* family: *MYB* genes based on B73 reference genome V5.0 and expression patterns.																
No.	Name	Gene ID (B73 V5)[1]	Chromosomal localization		Expression Patterns[2]											
			chr	pos	Root	Leaf	Stem and Sam	Internode	Tassel	Anther	Silk	Cob	Seed	Endosperm	Embryo	Pericarp
181	*ZmMYB181*	*Zm00001eb360850*	8	156716457	-	-	-	+	-	-	-	-	+	-	-	-
182	*ZmMYB182*	*Zm00001eb029300*	1	158672333	+	+	+	+	+	+	+	+	+	-	+	+
183	*ZmMYB183*	*Zm00001eb366540*	8	172367546	+	+	+	+	+	+	+	-	+	+	-	-
184	*ZmMYB184*	*Zm00001eb386370*	9	95456371	+	+	+	+	+	+	+	+	+	+	+	+
185	*ZmMYB185*	*Zm00001eb417170*	10	87045298	+	-	-	-	-	-	-	-	-	-	-	-
186	*ZmMYB186*	*Zm00001eb041450*	1	218413248	-	+	+	+	+	-	-	+	+	+	+	+
187	*ZmMYB187*	*Zm00001eb348760*	8	103804659	+	+	+	+	+	-	+	+	+	+	+	+
188	*ZmMYB188*	*Zm00001eb216040*	5	10665158	+	+	-	+	+	+	-	-		-	-	-
189	*ZmMYB189*	*Zm00001eb128770*	3	43511364	-	-	-	-	-	-	-	-	+	-	-	-
190	*ZmMYB190*	*Zm00001eb119340*	3	2231146	+	+	+	+	+	+	-	+	+	+	+	+
191	*ZmMYB191*	*Zm00001eb185920*	4	153013525	+	+	+	+	-	-	-	+	+	+	-	+
192	*ZmMYB192*	*Zm00001eb278680*	6	119938912	+	+	-	+	-	+	-	-	-	-	-	-
193	*ZmMYB193*	*Zm00001eb387370*	9	101707433	+	+	+	+	+	+	+	+	+	+	+	+
194	*ZmMYB194*	*Zm00001eb138640*	3	140028666	+	-	-	-	-	-	-	-	+	+	-	+
195	*ZmMYB195*	*Zm00001eb187810*	4	162986784	+	+	+	+	+	-	-	+	+	-	-	-
196	*ZmMYB196*	*Zm00001eb060980*	1	295423285	+	+	+	+	+	+	+	+	+	+	+	+

Notes: 1. Gene ID was based on Zm-B73-REFERENCE-NAM-5.0; 2. The expression information of maize *ZmMYB* genes was based on B73 RefGen_v3;1 and 2 retrieved from MaizeGDB (www.maizegdb.org, accessed on 22 March 2026) "+" and "−" indicate whether or not the gene was expressed in the corresponding tissue, and "+" was highlighted with gray background.
