# Supplementary material for: Genome-Wide Study of MYB Transcription Factors in Maize and Their Essential Roles in Male Fertility and Other Biological Processes
Source: Int J Mol Sci. 2026 Jun 27;27(13):5822. doi: 10.3390/ijms27135822 (PMC13360694; doi:10.3390/ijms27135822)
Supplement: Supplementary file 1 [file ijms-27-05822-s001.zip › Supplementary Table S2 Functional classifications of the reported main MYB transcription factors in Arabidopsis,rice,and their orthologs in maize-v2.pdf]

Table S2: Functional classifications of the reported main *MYB* transcription factors in *Arabidopsis*, rice, and their orthologs in maize.							
No.	Gene name	Gene ID	Gene Name in This Paper	Maize Orthologs	Expression Organs	Biological Functions	References
I. Anther development and male fertility							
1	*ZmMs9*	*Zm00001eb013670*	*ZmMYB175*	*Zm00001eb013670*	Anther	Male germ cells formation	[1]
1	*OsTDF1*	*Os03g0296000*	*ZmMYB175*	*Zm00001eb013670*	Tapetal cells and meiocytes	Tapetal development	[2]
1	*AtMYB35/AtTDF1*	*AT3G28470*	*ZmMYB175*	*Zm00001eb013670*	Tapetal cells and meiocytes	Tapetal development and degradation	[3]
2	*ZmMYB33-1*	*Zm00001eb369190*	*ZmMYB67*	*Zm00001eb369190*	Anther	Anther Development and Pollen Fertility	[4]
2	*OsCSA2*	*Os05g0490600*	*ZmMYB67*	*Zm00001eb369190*	Anther	Sugar Partitioning Required for Rice Pollen Development	[5]
2	*AtMYB65*	*AT3G11440*	*ZmMYB67*	*Zm00001eb369190*	Anther	Tapetal development and degradation	[6,7]
3	*ZmMYB33-2*	*Zm00001eb150140*	*ZmMYB147*	*Zm00001eb150140*	Anther	Anther Development and Pollen Fertility	[4]
3	*AtMYB33*	*AT5G06100*	*ZmMYB147*	*Zm00001eb150140*	Anther	Tapetal development and degradation	[6,7]
4	*ZmMYB84*	*Zm00001eb424660*	*ZmMYB170*	*Zm00001eb424660*	Tapetum and pollen grains	Pollen Development and the Regulation of Tapetal Programmed Cell Death	[8,9]
4	*OsMYB80*	*Os04g0470600*	*ZmMYB170*	*Zm00001eb424660*	Anther	Anther Development and Pollen Fertility	[10]
4	*AtMYB80*	*AT5G56110*	*ZmMYB170*	*Zm00001eb424660*	Anther	Anther Development and Pollen Fertility	[11]
5	*OsAID1*	*Os06g0181300*	*ZmMYB25*	*Zm00001eb271970*	Panicle and leaves	Anther dehiscence	[12]
6	*OsCSA*	*Os01g0274800*	*ZmMYB104*	*Zm00001eb128760*	Vascular Tissues and the Tapetum	Sugar Partitioning Required for Rice Pollen Development	[13]
7	*OsGAMYB*	*Os01g0812000*	*ZmMYB67*	*Zm00001eb369190*	Anther	Tapetal development and pollen grains formation	[14,15]
7	*OsGAMYB*	*Os01g0812000*	*ZmMYB147*	*Zm00001eb150140*	Anther	Tapetal development and pollen grains formation	[14,15]
8	*AtMYB21*	*AT3G27810*	*ZmMYB149*	*Zm00001eb158910*	Anther	Stamen Filament Growth and anther dehiscence	[16,17]
8	*AtMYB24*	*AT5G40350*	*ZmMYB149*	*Zm00001eb158910*	Anther	Stamen Filament Growth and anther dehiscence	[16,17]
8	*AtMYB57*	*AT3G01530*	*ZmMYB149*	*Zm00001eb158910*	Anther	Stamen Filament Growth and anther dehiscence	[16,17]

Table S2: Functional classifications of the reported main *MYB* transcription factors in *Arabidopsis*, rice, and their orthologs in maize.

9	*AtMYB108*	*AT3G06490*	*ZmMYB148*	*Zm00001eb028610*	stamens of flowers	Stamen Maturation	[18]
			ZmMYB172	*Zm00001eb111710*			
			ZmMYB172	*Zm00001eb130020*			
			ZmMYB158	*Zm00001eb136760*			
			ZmMYB142	*Zm00001eb165000*			
			ZmMYB77	*Zm00001eb339810*			
			ZmMYB178	*Zm00001eb354750*			
			ZmMYB185	*Zm00001eb417170*			
			ZmMYB54	*Zm00001eb417190*			
10	*AtMYB26/MS35*	*AT3G13890*	*ZmMYB113*	*Zm00001eb155190*	Buds and flower	Anther dehiscence	[19]
11	*AtMYB4*	*AT4G38620*	*ZmMYB82*	*Zm00001eb103730*	Anther	Tapetal development and pollen grains formation	[20]
12	*AtMYB101*	*AT2G32460*	*ZmMYB95*	*Zm00001eb219740*	mature pollen	Pollen Tube-Synergid Interaction	[21]
			ZmMYB159	*Zm00001eb224600*			
13	*AtMYB120*	*AT5G55020*	*ZmMYB179*	*Zm00001eb273750*			
14	*AtMYB125/AtDUO1*	*AT3G60460*	*ZmMYB76*	*Zm00001eb035590*	Male germline	Male germ cells formation	[22]
			ZmMYB129	*Zm00001eb074810*			
15	*AtMYB81*	*AT2G26960*	*ZmMYB119*	*Zm00001eb273570*	Anther	Male germ cells formation	[23]
			ZmMYB120	*Zm00001eb273580*			
			ZmMYB184	*Zm00001eb386370*			
II. Vegetative organ and seed development							
1	*ZmMYBR29*	*Zm00001eb108630*	*ZmMYBR29*	-	Seed	modulate grain size	[24]
2	*OsMYB1-R1*	*Os04g0583900*	*ZmMYB20*	*Zm00001eb428070*	Developing grain	Regulates grain yield	[25]
3	*OsMYB2P-1*	*Os05g0140100*	*ZmMYB30*	*Zm00001eb354510*	Root	Regulates root configuration	[26]
4	*OsMYB21*	*Os11g0684000*	*ZmMYB100*	*Zm00001eb028610*	Inflorescence/Seed	Promotes seed fall	[27]
5	*OsMYBS1*	*Os01g0524500*	*ZmMYB187*	*Zm00001eb129010*	leaf	modulating leaf morphology	[28]
			ZmMYB189	*Zm00001eb332640*			
			ZmMYB181	*Zm00001eb442430*			
6	*AtMYB103*	*AT5G56110*	*ZmMYB170*	*Zm00001eb424660*	Trichomes	Trichome development	[9]
7	*AtMYB56*	*AT5G17800*	*ZmMYB189*	*Zm00001eb128770*	Developing seed	Regulate seed size	[29]
8	*AtMYB83*	*AT3G08500*	*ZmMYB109*	*Zm00001eb410950*	Root hairs	Response root hair growth	[30]
9	*AtMYB5*	*AT3G13540*	*ZmMYB181*	*Zm00001eb360850*	Trichomes and developing seed coats	Control outer seed coat differentiation	[31]
III. Biotic and abiotic stress response							

Table S2: Functional classifications of the reported main *MYB* transcription factors in *Arabidopsis*, rice, and their orthologs in maize.

1	*ZmMYB30*	*Zm00001eb095690*	*ZmMYB111*	*Zm00001eb095690*	Constitutive expression	Promote salt-stress tolerance	[32]
	AtMYB44	*AT5G67300*			Constitutive expression	Promote salt-stress tolerance	[33]
	AtMYB77	*AT3G50060*			shoot and root	Involved in UV-B and auxin response	[34]
2	*AtMYB7*	*AT2G16720*	*ZmMYB187*	*Zm00001eb348760*	mature seeds and pollen	Response to salt UV-B, ABA, salt stress	[35,36]
	AtMYB3	*AT1G22640*			Various tissues	Response to salt stress	[37]
3	*ZmMYB39*	*Zm00001eb185160*	*ZmMYB39*	-	Roots， stems, leaves, Flower, and tassels,	Regulate heat stress response	[38]
4	*ZmMYBR24*	*Zm00001eb337410*	*ZmGLK68*	-	roots	Response to salt and low-temperature stress	[39]
5	*ZmMYB104*	*Zm00001eb074810*	*ZmMYB104*	-	Young seedling	Response to drought stresses	[40]
6	*ZmMYB31*	*Zm00001eb103730*	*ZmMYB82*	-	Leaf and embryo	Sensitivity to UV radiation	[41]
7	*ZmMYB-R1*	*Zm00001eb256690*	*ZmMYB8*	-	Leaf, stem and root	Responsible for drought tolerance	[42]
8	*ZmMYB48*	*Zm00001eb028820*	*ZmMYB141*	-	Tassel and leaf	Participates in the drought stress response and seed germination	[43]
9	*ZmMYB121*	*Zm00001eb045780*	*ZmMYB121*		Leaf	Response to drought stresses	[44]
10	*OsFLP*	*Os07g0627300*	*ZmMYB7*	Zm00001eb109820	young shoots、leaf and flower	Response to drought and salt stresses	[45]
11	*OsMYBR57*	*Os06g0181300*	*ZmMYBR117*	Zm00001eb111250	Leaf	Response to drought stresses	[46]
			ZmMYBR98	Zm00001eb271970			
			ZmMYBR130	Zm00001eb374860			
12	*OsMYB-R1*	*Os06g0670300*	*ZmGLK32*	Zm00001eb390020	root	Response to drought and chromium stresses	[47]
			ZmGLK31	Zm00001eb274260			
13	*OsMYB61*	*Os05g0140100*	*ZmMYB30*	Zm00001eb354510	Leaf and seedling	Response to heat stress	[48]
			ZmMYB149	Zm00001eb417620			
14	*OsMYB110*	*Os10g0478300*	*ZmMYB150*	*Zm00001eb045780*	Leaf and root	Positive regulator of resistance against fungal and bacterial pathogens	[49]

Table S2: Functional classifications of the reported main *MYB* transcription factors in *Arabidopsis*, rice, and their orthologs in maize.							
15	*OsMYB30*	*Os02g0624300*	*ZmMYB134*	*Zm00001eb099830*	Leaf and root	Prevents fungal penetration of rice leaves and confers brown planthopper resistance	[50,51]
			ZmMYB135	*Zm00001eb313580*			
16	*AtMYB74*	*AT4G05100*	*ZmMYB162*	*Zm00001eb106790*	Constitutive expression	Response to salt stress	[52]
			ZmMYB68	*Zm00001eb324070*			
17	*AtMYB52*	*AT1G17950*	*ZmMYB98*	*Zm00001eb055540*	Roots and siliques	Response to ABA and drought stresses	[53]
			ZmMYB188	*Zm00001eb216040*			
18	*AtMYB71*	*AT3G24310*	*ZmMYB87*	*Zm00001eb003470*	Flower and root	Regulates ABA response	[54]
			ZmMYB73	*Zm00001eb184890*			
			ZmMYB66	*Zm00001eb247370*			
			ZmMYB115	*Zm00001eb402890*			
19	*AtMYB5*	*AT3G13540*	*ZmMYB181*	*Zm00001eb360850*	Seed	Regulate heat stress response	[55]
20	*AtBOS1/AtMYB108*	*AT3G06490*	*ZmMYB148*	*Zm00001eb028610*	Flower and leaf	Required for Biotic and Abiotic Stress Responses	[56]
			ZmMYB172	*Zm00001eb111710*			
			ZmMYB172	*Zm00001eb130020*			
			ZmMYB158	*Zm00001eb136760*			
			ZmMYB142	*Zm00001eb165000*			
			ZmMYB77	*Zm00001eb339810*			
			ZmMYB178	*Zm00001eb354750*			
			ZmMYB185	*Zm00001eb417170*			
			ZmMYB54	*Zm00001eb417190*			
			ZmMYB148	*Zm00001eb028610*			
21	*AtMYB96*	*AT5G62470*	*ZmMYB89*	*Zm00001eb328280*	Flower and leaf	MYB96-mediated abscisic acid signals induce pathogen resistance	[57]
22	*AtMYB4*	*AT4G38620*	*ZmMYB105*	*Zm00001eb040070*	Leaf	Regulate plant sensitivity to UV-B	[58]
			ZmMYB82	*Zm00001eb103730*			
23	*AtMYB12*	*AT2G47460*	*ZmMYB106*	*Zm00001eb014260*	seedling	Response to drought and oxidative stresses	[59-61]
			ZmMYB64	*Zm00001eb396390*			
			ZmMYB107	*Zm00001eb014280*			
			ZmMYB90	*Zm00001eb014290*			
			ZmMYB108	*Zm00001eb014320*			
24	*AtMYB101*	*AT2G32460*	*ZmMYB95*	*Zm00001eb219740*	Seed	Response to ABA stress	[62]
			ZmMYB159	*Zm00001eb224600*			

Table S2: Functional classifications of the reported main *MYB* transcription factors in *Arabidopsis*, rice, and their orthologs in maize.							
			ZmMYB179	*Zm00001eb273750*			
25	*AtMYB20*	*AT1G66230*	*ZmMYB130*<br>*ZmMYB88*	*Zm00001eb041860*<br>*Zm00001eb190160*	Veins of leaf and stems	Response to drought and salt stresses	[63,64]
			ZmMYB81	*Zm00001eb280660*			
			ZmMYB165	*Zm00001eb312120*			
26	*AtMYB42*	*AT4G12350*	*ZmMYB145*	*Zm00001eb202670*	Seedlings, leaves and roots	Response to salt ABA, salt stress，seed germination and root growth	[65] [66]
			ZmMYB127	*Zm00001eb319620*			
27	*AtMYB121*	*AT3G30210*	*ZmMYB120*	Zm00001eb003470	Seedlings, leaves and roots	Response to ABA, salt stress，seed germination and root growth	[66]
			ZmMYB144	Zm00001eb184890			
			ZmMYB86	Zm00001eb247370			
			ZmMYB165	Zm00001eb402890			
			ZmMYB221	Zm00001eb426280			
28	*AtMYB11*	*AT3G62610*	*ZmMYB55*<br>*ZmMYB201*	Zm00001eb014260<br>Zm00001eb014280	Guard cells, leaf epidermis	Response to oxidative stresses	[67]
			ZmMYB3	Zm00001eb014290			
			ZmMYB224	Zm00001eb014320			
			ZmMYB178	Zm00001eb014350			
			ZmMYB217	Zm00001eb014430			
			ZmMYB95	Zm00001eb340000			
			ZmMYB154	Zm00001eb396390			
29	*AtMYBS1*	*AT2G26960*	*ZmMYB189*	Zm00001eb332640	Whole plant	Response to heat stress	[68]
			ZmMYB181	Zm00001eb442430			
			ZmMYB187	Zm00001eb129010			
30	*AtMYB103*	*AT1G63910*	*ZmMYB92*	Zm00001eb416400	Root and hemicellulose	Response to aluminum stress	[69]
31	*AtMYB15*	*AT3G23250*	*ZmMYB121*	Zm00001eb045780	Leaf	Response to pathogen infection	[70]
			ZmMYB39	Zm00001eb185160			
			ZmMYB218	Zm00001eb221780			
			ZmMYB41<br>*ZmMYB132*	Zm00001eb247570<br>Zm00001eb426670			
32	*AtMYB25*	*AT2G39880*	*ZmMYBR46*	Zm00001eb147880	roots, young leaf, anthers, and the extremities of siliques	Response to ABA, osmotic and salt stress	[71]
			ZmMYBR36	Zm00001eb367660			

Table S2: Functional classifications of the reported main *MYB* transcription factors in *Arabidopsis*, rice, and their orthologs in maize.

33	*AtMYB37*	*AT5G23000*	*ZmMYB193*	Zm00001eb086570	leaf and root tips	Response to salt and drought stresses	[72]
34	*AtMYB32*	*AT4G34990*	*ZmMYB38*	Zm00001eb040070	root	Response to drought stresses	[73]
			ZmMYB31	Zm00001eb103730			
			ZmMYB11	Zm00001eb332030			
35	*AtMYB30*	*AT3G28910*	*ZmMYB83*	Zm00001eb041320	root	Response to Fe homeostasis	[74]
			ZmMYB33	Zm00001eb041330			
			ZmMYB35	Zm00001eb099570			
			ZmMYB70	Zm00001eb109860			
			ZmMYB162	Zm00001eb312600			
			ZmMYB52	Zm00001eb392230			
IV. Regulation of primary and secondary metabolism							
1	*ZmMYB14*	*Zm00001eb321920*	*ZmMYB167*	-	Seed, root, stem, anther, pericarp, and embryo	Regulates starch biosynthesis	[75]
2	*ZmMYB71*	*Zm00001eb286270*	*ZmMYB71*	-	Endosperm, embryo and stem	Regulates starch biosynthesis	[76]
3	*ZmMYB92*	*Zm00001eb416400*	*ZmMYB92*	-	Stems and leaf veins	Secondary Cell Wall Biosynthesis	[77]
4	*ZmMYB155*	*Zm000001eb415180*	*ZmMYB155*	-	Endosperm	Regulates starch biosynthesis	[78]
5	*ZmMYB127*	*Zm00001eb139600*	*ZmMYB127*	-	Endosperm	Regulates starch, zein and auxin synthesis pathways biosynthesis	[79,80]
6	*OsRLM1*	*Os05g0543600*	*ZmMYB171*	*Zm00001eb294560*	Young seedling and stem tissue	Regulates the Development of Secondary Cell Wall	[81]
			ZmMYB136	*Zm00001eb342580*			
7	*Os^{C1PLSr}*	*Os05g0553400*	*ZmMYB130*	*Zm00001eb295060*	leaf sheath	Regulates anthocyanin synthesis	[82]
8	*OsP1*	*Os03g19120*	*ZmMYB55*	*Zm00001eb014260*	leaf	Regulates anthocyanin synthesis	[83]
			ZmMYB201	*Zm00001eb014280*			
			ZmMYB3	*Zm00001eb014290*			
			ZmMYB224	*Zm00001eb014320*			
			ZmMYB178	*Zm00001eb014350*			
			ZmMYB217	*Zm00001eb014430*			
			ZmMYB154	*Zm00001eb396390*			

Table S2: Functional classifications of the reported main *MYB* transcription factors in *Arabidopsis*, rice, and their orthologs in maize.							
9	*OsMYB1*	*Os02g0187700*	*ZmMYB50*	*Zm00001eb234690*	Leaf and pericarps	Regulates anthocyanin synthesis	[84]
10	*AtMYB58*<br>*AtMYB63*	*AT1G16490*<br>*AT1G79180*	*ZmMYB70*	*Zm00001eb072200*	Fibers and Vessels	Lignin biosynthesis	[85]
			ZmMYB123	*Zm00001eb430000*			
			ZmMYB124	*Zm00001eb430010*			
			ZmMYB195	*Zm00001eb187810*			
11	*AtMYB51*	*AT1G18570*	*ZmMYB132*	*Zm00001eb033610*	Hypocotyl, cotyledons, roots of young seedlings, leaves, young flowers, and the abscission zone of fully developed siliques	Indolic glucosinolate biosynthesis	[86]
			ZmMYB62	*Zm00001eb199170*			
			ZmMYB156	*Zm00001eb255300*			
			ZmMYB126	*Zm00001eb268770*			
			ZmMYB74	*Zm00001eb379110*			
			ZmMYB102	*Zm00001eb429940*			
12	*AtMYB85*	*AT4G22680*	*ZmMYB145*	*Zm00001eb202670*	secondary xylem	Secondary Cell Wall Biosynthesis	[87]
	AtMYB42	*AT4G12350*	*ZmMYB127*	*Zm00001eb319620*			
	AtMYB52	*AT1G17950*	*ZmMYB98*	*Zm00001eb055540*			
	AtMYB54	*AT1G73410*	*ZmMYB188*	*Zm00001eb216040*			
	AtMYB69	*AT4G33450*	*ZmMYB97*	*Zm00001eb237460*			
	AtMYB20	*AT1G66230*	*ZmMYB130*	*Zm00001eb041860*			
			ZmMYB88	*Zm00001eb190160*			
			ZmMYB81	*Zm00001eb280660*			
			ZmMYB165	*Zm00001eb312120*			

References

1. Greyson, R.I.; Walden, D.B.; Cheng, P.C. LM, TEM AND SEM OBSERVATIONS OF ANTHER DEVELOPMENT IN THE GENIC MALE-STERILE (ms 9) MUTANT OF CORN ZEA MAYS. *Canadian Journal of Genetics and Cytology* **1980**, *22*, 153-166, doi:10.1139/g80-020.
2. Cai, C.-F.; Zhu, J.; Lou, Y.; Guo, Z.-L.; Xiong, S.-X.; Wang, K.; Yang, Z.-N. The functional analysis of OsTDF1 reveals a conserved genetic pathway for tapetal development between rice and Arabidopsis. *Science Bulletin* **2015**, *60*, 1073-1082, doi:https://doi.org/10.1007/s11434-015-0810-3.
3. Zhu, J.; Chen, H.; Li, H.; Gao, J.-F.; Jiang, H.; Wang, C.; Guan, Y.-F.; Yang, Z.-N. Defective in Tapetal Development and Function 1 is essential for anther development and tapetal function for microspore maturation in Arabidopsis. *The Plant Journal* **2008**, *55*, 266-277, doi:https://doi.org/10.1111/j.1365-313X.2008.03500.x.
4. Jiang, Y.; An, X.; Li, Z.; Yan, T.; Zhu, T.; Xie, K.; Liu, S.; Hou, Q.; Zhao, L.; Wu, S.; et al. CRISPR/Cas9-based discovery of maize transcription factors regulating male sterility and their functional conservation in plants. *Plant Biotechnol J* **2021**, *19*, 1769-1784, doi:10.1111/pbi.13590.
5. Wang, D.; Li, J.; Sun, L.; Hu, Y.; Yu, J.; Wang, C.; Zhang, F.; Hou, H.; Liang, W.; Zhang, D. Two rice MYB transcription factors maintain male fertility in response to photoperiod by modulating sugar partitioning. *New Phytologist* **2021**, *231*, 1612-1629, doi:https://doi.org/10.1111/nph.17512.
6. Millar, A.A.; Gubler, F. The Arabidopsis GAMYB-Like Genes, MYB33 and MYB65, Are MicroRNA-Regulated Genes That Redundantly Facilitate Anther Development. *The Plant Cell* **2005**, *17*, 705-721, doi:10.1105/tpc.104.027920.
7. Liu, B.; De Storme, N.; Geelen, D. Gibberellin Induces Diploid Pollen Formation by Interfering with Meiotic Cytokinesis. *Plant Physiology* **2016**, *173*, 338-353, doi:10.1104/pp.16.00480.
8. Phan, H.A.; Iacuone, S.; Li, S.F.; Parish, R.W. The MYB80 Transcription Factor Is Required for Pollen Development and the Regulation of Tapetal Programmed Cell Death in Arabidopsis thaliana *The Plant Cell* **2011**, *23*, 2209-2224, doi:10.1105/tpc.110.082651.
9. Higginson, T.; Li, S.F.; Parish, R.W. AtMYB103 regulates tapetum and trichome development in Arabidopsis thaliana. *The Plant Journal* **2003**, *35*, 177-192, doi:https://doi.org/10.1046/j.1365-313X.2003.01791.x.
10. Pan, X.; Yan, W.; Chang, Z.; Xu, Y.; Luo, M.; Xu, C.; Chen, Z.; Wu, J.; Tang, X. OsMYB80 Regulates Anther Development and Pollen Fertility by Targeting Multiple Biological Pathways. *Plant and Cell Physiology* **2020**, *61*, 988-1004, doi:10.1093/pcp/pcaa025.
11. Jiang, Y.; An, X.; Li, Z.; Yan, T.; Zhu, T.; Xie, K.; Liu, S.; Hou, Q.; Zhao, L.; Wu, S.; et al. CRISPR/Cas9-based discovery of maize transcription factors regulating male sterility and their functional conservation in plants. *Plant Biotechnology Journal* **2021**, *19*, 1769-1784, doi:https://doi.org/10.1111/pbi.13590.
12. Zhu, Q.-H.; Ramm, K.; Shivakkumar, R.; Dennis, E.S.; Upadhyaya, N.M. The ANTHER INDEHISCENCE1 Gene Encoding a Single MYB Domain Protein Is Involved in Anther Development in Rice. *Plant Physiology* **2004**, *135*, 1514-1525, doi:10.1104/pp.104.041459.
13. Zhang, H.; Liang, W.; Yang, X.; Luo, X.; Jiang, N.; Ma, H.; Zhang, D. Carbon Starved Anther Encodes a MYB Domain Protein That Regulates Sugar Partitioning Required for Rice Pollen Development *The Plant Cell* **2010**, *22*, 672-689, doi:10.1105/tpc.109.073668.
14. Kaneko, M.; Inukai, Y.; Ueguchi-Tanaka, M.; Itoh, H.; Izawa, T.; Kobayashi, Y.; Hattori, T.; Miyao, A.; Hirochika, H.; Ashikari, M.; et al. Loss-of-Function Mutations of the Rice GAMYB Gene Impair α-Amylase Expression in Aleurone and Flower Development. *The Plant Cell* **2004**, *16*, 33-44, doi:10.1105/tpc.017327.
15. Aya, K.; Ueguchi-Tanaka, M.; Kondo, M.; Hamada, K.; Yano, K.; Nishimura, M.; Matsuoka, M. Gibberellin Modulates Anther Development in Rice via the Transcriptional Regulation of GAMYB. *The Plant Cell* **2009**, *21*, 1453-1472, doi:10.1105/tpc.108.062935.
16. Mandaokar, A.; Thines, B.; Shin, B.; Markus Lange, B.; Choi, G.; Koo, Y.J.; Yoo, Y.J.; Choi, Y.D.; Choi, G.; Browse, J. Transcriptional regulators of stamen development in Arabidopsis identified by transcriptional profiling. *The Plant Journal* **2006**, *46*, 984-1008, doi:https://doi.org/10.1111/j.1365-313X.2006.02756.x.
17. Cheng, H.; Song, S.; Xiao, L.; Soo, H.M.; Cheng, Z.; Xie, D.; Peng, J. Gibberellin Acts through Jasmonate to Control the Expression of MYB21, MYB24, and MYB57 to Promote Stamen Filament Growth in Arabidopsis. *PLOS Genetics* **2009**, *5*,

e1000440, doi:10.1371/journal.pgen.1000440.
18. Mandaokar, A.; Browse, J. MYB108 Acts Together with MYB24 to Regulate Jasmonate-Mediated Stamen Maturation in Arabidopsis. *Plant Physiology* **2008**, *149*, 851-862, doi:10.1104/pp.108.132597.
19. Steiner-Lange, S.; Unte, U.S.; Eckstein, L.; Yang, C.; Wilson, Z.A.; Schmelzer, E.; Dekker, K.; Saedler, H. Disruption of Arabidopsis thaliana MYB26 results in male sterility due to non-dehiscent anthers. *The Plant Journal* **2003**, *34*, 519-528, doi:https://doi.org/10.1046/j.1365-313X.2003.01745.x.
20. Preston, J.; Wheeler, J.; Heazlewood, J.; Li, S.F.; Parish, R.W. AtMYB32 is required for normal pollen development in Arabidopsis thaliana. *The Plant Journal* **2004**, *40*, 979-995, doi:https://doi.org/10.1111/j.1365-313X.2004.02280.x.
21. Liang, Y.; Tan, Z.-M.; Zhu, L.; Niu, Q.-K.; Zhou, J.-J.; Li, M.; Chen, L.-Q.; Zhang, X.-Q.; Ye, D. MYB97, MYB101 and MYB120 Function as Male Factors That Control Pollen Tube-Synergid Interaction in Arabidopsis thaliana Fertilization. *PLOS Genetics* **2013**, *9*, e1003933, doi:10.1371/journal.pgen.1003933.
22. Rotman, N.; Durbarry, A.; Wardle, A.; Yang, W.C.; Chaboud, A.; Faure, J.-E.; Berger, F.; Twell, D. A Novel Class of MYB Factors Controls Sperm-Cell Formation in Plants. *Current Biology* **2005**, *15*, 244-248, doi:10.1016/j.cub.2005.01.013.
23. Oh, S.-A.; Hoai, T.N.T.; Park, H.-J.; Zhao, M.; Twell, D.; Honys, D.; Park, S.-K. MYB81, a microspore-specific GAMYB transcription factor, promotes pollen mitosis I and cell lineage formation in Arabidopsis. *The Plant Journal* **2020**, *101*, 590-603, doi:https://doi.org/10.1111/tpj.14564.
24. Wu, J.W.; Wang, X.Y.; Yan, R.Y.; Zheng, G.M.; Zhang, L.; Wang, Y.; Zhao, Y.J.; Wang, B.H.; Pu, M.L.; Zhang, X.S.; et al. A MYB-related transcription factor ZmMYBR29 is involved in grain filling. *BMC PLANT BIOLOGY* **2024**, *24*, doi:10.1186/s12870-024-05163-9.
25. Duan, M.; Huang, P.; Yuan, X.; Chen, H.; Huang, J.A.-O.; Zhang, H. CMYB1 encoding a MYB transcriptional activator is involved in abiotic stress and circadian rhythm in rice. *ScientificWorldJournal. 2014;2014:178038. doi: 10.1155/2014/178038. Epub 2014 Apr 1.*
26. Dai, X.; Wang Y Fau - Yang, A.; Yang A Fau - Zhang, W.-H.; Zhang, W.H. OsMYB2P-1, an R2R3 MYB transcription factor, is involved in the regulation of phosphate-starvation responses and root architecture in rice. *Plant Physiol. 159*, 169-183. doi: 110.1104/pp.1112.194217. Epub 192012 Mar 194216.
27. Ning, J.; He, W.; Wu, L.; Chang, L.; Hu, M.; Fu, Y.; Liu, F.A.-O.; Sun, H.A.-O.; Gu, P.; Ndjiondjop, M.N.; et al. The MYB transcription factor Seed Shattering 11 controls seed shattering by repressing lignin synthesis in African rice. *Plant Biotechnol J. 21*, 931-942. doi: 910.1111/pbi.14004. Epub 12023 Jan 14026.
28. Ma, Y.H.; Zhang, J.; Liang, K.; Huang, A.Q.; Liu, J.S.; Li, W.W.; Sun, B.; Zhao, Y. Overexpression of OsMYBS1 affect leaf morphology, photosynthesis, and agronomic performance in rice. *FRONTIERS IN PLANT SCIENCE* **2025**, *16*, doi:10.3389/fpls.2025.1653514.
29. Zhang, Y.; Liang, W.; Shi, J.; Xu, J.; Zhang, D. MYB56 Encoding a R2R3 MYB Transcription Factor Regulates Seed Size in Arabidopsis thaliana. *Journal of Integrative Plant Biology* **2013**, *55*, 1166-1178, doi:https://doi.org/10.1111/jipb.12094.
30. Cheng, X.; Zhen, H.P.; Wang, J.S.; Li, M.Y.; Liu, S.H.; Gao, Y.; Zhang, N.; Fu, Y.; Wang, C. MYB83 plays an important negative role in ethylene-mediated root hair growth and involves in plant tolerance three major nutrient deficiencies. *PLANT JOURNAL* **2026**, *126*, doi:10.1111/tpj.70882.
31. Gonzalez, A.; Mendenhall, J.; Huo, Y.; Lloyd, A. TTG1 complex MYBs, MYB5 and TT2, control outer seed coat differentiation. *Developmental Biology* **2009**, *325*, 412-421, doi:https://doi.org/10.1016/j.ydbio.2008.10.005.
32. Chen, Y.H.; Cao, Y.Y.; Wang, L.J.; Li, L.M.; Yang, J.; Zou, M.X. Identification of MYB transcription factor genes and their expression during abiotic stresses in maize. *BIOLOGIA PLANTARUM* **2018**, *62*, 222-230, doi:10.1007/s10535-017-0756-1.
33. Liu, R.; Lü, B.; Wang, X.; Zhang, C.; Zhang, S.; Qian, J.; Chen, L.; Shi, H.; Dong, H. Thirty-seven transcription factor genes differentially respond to a harpin protein and affect resistance to the green peach aphid in Arabidopsis. *Journal of Biosciences* **2010**, *35*, 435-450, doi:10.1007/s12038-010-0049-8.
34. Yang, Y.; Zhang, L.; Chen, P.; Liang, T.; Li, X.; Liu, H. UV-B photoreceptor UVR8 interacts with MYB73/MYB77 to regulate auxin responses and lateral root development. *The EMBO Journal* **2020**, *39*, e101928, doi:https://doi.org/10.15252/embj.2019101928.
35. Fornalé, S.; Lopez, E.; Salazar-Henao, J.E.; Fernández-Nohales, P.; Rigau, J.; Caparros-Ruiz, D. AtMYB7, a New Player in the Regulation of UV-Sunscreens in Arabidopsis thaliana. *Plant and Cell Physiology* **2014**, *55*, 507-516, doi:10.1093/pcp/pct187.
36. KIM, J.H.; HYUN, W.Y.; NGUYEN, H.N.; JEONG, C.Y.; XIONG, L.; HONG, S.-W.; LEE, H. AtMyb7, a subgroup 4 R2R3 Myb,

negatively regulates ABA-induced inhibition of seed germination by blocking the expression of the bZIP transcription factor ABI5. *Plant, Cell & Environment* **2015**, *38*, 559-571, doi:https://doi.org/10.1111/pce.12415.
37. Kim, D.; Jeon, S.J.; Yanders, S.; Park, S.C.; Kim, H.S.; Kim, S. MYB3 plays an important role in lignin and anthocyanin biosynthesis under salt stress condition in Arabidopsis. *Plant Cell Reports* **2022**, *41*, 1549-1560, doi:10.1007/s00299-022-02878-7.
38. Ren, Z.; Zhang, P.; Su, H.; Xie, X.; Shao, J.; Ku, L.; Tian, Z.; Deng, D.; Wei, L. Regulatory mechanisms used by ZmMYB39 to enhance drought tolerance in maize (Zea mays) seedlings. *Plant Physiology and Biochemistry* **2024**, *211*, 108696, doi:https://doi.org/10.1016/j.plaphy.2024.108696.
39. Bao, L.; Sun, W.; Wang, J.; Zhou, Y.; Wang, J.; Wang, Q.; Sun, D.; Lin, H.; Fan, J.; Zhou, Y.; et al. The Transcription Factor ZmMYBR24 Gene Is Involved in a Variety of Abiotic Stresses in Maize (Zea mays L.). *Plants* **2025**, *14*, 2054, doi:10.3390/plants14132054.
40. Zhang, H.; Wang, Q.Y.; Zhou, T.; Qiu, X.Q.; Ma, C.H.; Zhang, J.H.; Sahito, J.H.; Liu, Y.; Zhao, J.W.; Li, J.; et al. ZmMYB104 Enhances Heat-Stress Tolerance by Activating ZmCAT2 Expression in Maize. *PHYSIOLOGIA PLANTARUM* **2025**, *177*, doi:10.1111/ppl.70478.
41. Fornalé, S.; Shi, X.; Chai, C.; Encina, A.; Irar, S.; Capellades, M.; Fuguet, E.; Torres, J.-L.; Rovira, P.; Puigdomènech, P.; et al. ZmMYB31 directly represses maize lignin genes and redirects the phenylpropanoid metabolic flux. *The Plant Journal* **2010**, *64*, 633-644, doi:https://doi.org/10.1111/j.1365-313X.2010.04363.x.
42. Liu, L.; Hao, Z.; Weng, J.; Li, M.; Zhang, D.; Bai, L.; Wang, L.; Li, X.; Zhang, S. Identification of drought-responsive genes by cDNA-amplified fragment length polymorphism in maize. *Annals of Applied Biology* **2012**, *161*, 203-213, doi:https://doi.org/10.1111/j.1744-7348.2012.00565.x.
43. Wang, Y.; Wang, Q.; Liu, M.; Bo, C.; Wang, X.; Ma, Q.; Cheng, B.; Cai, R. Overexpression of a maize MYB48 gene confers drought tolerance in transgenic arabidopsis plants. *Journal of Plant Biology* **2017**, *60*, 612-621, doi:10.1007/s12374-017-0273-y.
44. Lian, Y.Y.; Yang, S.P.; Tian, T.; Yang, Z.R.; Liu, S.X.; Fu, X.M.; Liu, C.Y.; Zhu, T.F.; Wang, Y.J.; Bai, Y.T.; et al. Natural variation in ZmDapF1 enhances maize drought resilience. *NATURE PLANTS* **2025**, *11*, doi:10.1038/s41477-025-02141-3.
45. Qu, X.; Zou, J.; Wang, J.; Yang, K.; Wang, X.; Le, J. A Rice R2R3-Type MYB Transcription Factor OsFLP Positively Regulates Drought Stress Response via OsNAC. *International Journal of Molecular Sciences* **2022**, *23*, 5873, doi:10.3390/ijms23115873.
46. Yang, L.; Chen, Y.; Xu, L.; Wang, J.; Qi, H.; Guo, J.; Zhang, L.; Shen, J.; Wang, H.; Zhang, F.; et al. The OsFTIP6-OsHB22-OsMYBR57 module regulates drought response in rice. *Molecular Plant* **2022**, *15*, 1227-1242, doi:10.1016/j.molp.2022.06.003.
47. Tiwari, P.; Indoliya, Y.; Chauhan, A.S.; Pande, V.; Chakrabarty, D. Over-expression of rice R1-type MYB transcription factor confers different abiotic stress tolerance in transgenic Arabidopsis. *Ecotoxicology and Environmental Safety* **2020**, *206*, 111361, doi:https://doi.org/10.1016/j.ecoenv.2020.111361.
48. Zhao, S.M.; Ma, Y.Q.; Ding, Y.; Dong, G.R.; Liu, C.L.; Ma, X.M.; Hou, B.K. Rice glycosyltransferase UGT706F1 functions in heat tolerance through glycosylating flavonoids under the regulation of transcription factor MYB61. *PLANT JOURNAL* **2025**, *121*, doi:10.1111/tpj.17252.
49. Kishi-Kaboshi, M.; Seo, S.; Takahashi, A.; Hirochika, H. The MAMP-Responsive MYB Transcription Factors MYB30, MYB55 and MYB110 Activate the HCAA Synthesis Pathway and Enhance Immunity in Rice. *Plant and Cell Physiology* **2018**, *59*, 903-915, doi:10.1093/pcp/pcy062.
50. Li, W.; Wang, K.; Chern, M.; Liu, Y.; Zhu, Z.; Liu, J.; Zhu, X.; Yin, J.; Ran, L.; Xiong, J.; et al. Sclerenchyma cell thickening through enhanced lignification induced by OsMYB30 prevents fungal penetration of rice leaves. *New Phytologist* **2020**, *226*, 1850-1863, doi:https://doi.org/10.1111/nph.16505.
51. He, J.; Liu, Y.; Yuan, D.; Duan, M.; Liu, Y.; Shen, Z.; Yang, C.; Qiu, Z.; Liu, D.; Wen, P.; et al. An R2R3 MYB transcription factor confers brown planthopper resistance by regulating the phenylalanine ammonia-lyase pathway in rice. *Proceedings of the National Academy of Sciences* **2020**, *117*, 271-277, doi:10.1073/pnas.1902771116.
52. Xu, R.; Wang, Y.; Zheng, H.; Lu, W.; Wu, C.; Huang, J.; Yan, K.; Yang, G.; Zheng, C. Salt-induced transcription factor MYB74 is regulated by the RNA-directed DNA methylation pathway in Arabidopsis. *Journal of Experimental Botany* **2015**, *66*, 5997-6008, doi:10.1093/jxb/erv312.
53. Park, M.Y.; Kang, J.-y.; Kim, S.Y. Overexpression of AtMYB52 confers ABA hypersensitivity and drought tolerance. *Molecules and Cells* **2011**, *31*, 447-454, doi:10.1007/s10059-011-0300-7.
54. Cheng, Y.; Ma, Y.; Zhang, N.; Lin, R.; Yuan, Y.; Tian, H.; Hussain, S.; Chen, S.; Yang, W.; Cai, L.; et al. The R2R3 MYB

Transcription Factor MYB71 Regulates Abscisic Acid Response in Arabidopsis. *Plants* **2022**, *11*, 1369.

55. Jacob, P.; Brisou, G.; Dalmais, M.; Thévenin, J.; van der Wal, F.; Latrasse, D.; Suresh Devani, R.; Benhamed, M.; Dubreucq, B.; Boualem, A.; et al. The Seed Development Factors TT2 and MYB5 Regulate Heat Stress Response in Arabidopsis. *Genes* **2021**, *12*, 746.
56. Mengiste, T.; Chen, X.; Salmeron, J.; Dietrich, R. The BOTRYTIS SUSCEPTIBLE1 Gene Encodes an R2R3MYB Transcription Factor Protein That Is Required for Biotic and Abiotic Stress Responses in Arabidopsis. *The Plant Cell* **2003**, *15*, 2551-2565, doi:10.1105/tpc.014167.
57. Seo, P.J.; Park, C.-M. MYB96-mediated abscisic acid signals induce pathogen resistance response by promoting salicylic acid biosynthesis in Arabidopsis. *New Phytologist* **2010**, *186*, 471-483, doi:https://doi.org/10.1111/j.1469-8137.2010.03183.x.
58. Zhao, J.; Zhang, W.; Zhao, Y.; Gong, X.; Guo, L.; Zhu, G.; Wang, X.; Gong, Z.; Schumaker, K.S.; Guo, Y. SAD2, an Importin β-Like Protein, Is Required for UV-B Response in Arabidopsis by Mediating MYB4 Nuclear Trafficking. *The Plant Cell* **2007**, *19*, 3805-3818, doi:10.1105/tpc.106.048900.
59. Wang, F.; Kong, W.; Wong, G.; Fu, L.; Peng, R.; Li, Z.; Yao, Q. AtMYB12 regulates flavonoids accumulation and abiotic stress tolerance in transgenic Arabidopsis thaliana. *Molecular Genetics and Genomics* **2016**, *291*, 1545-1559, doi:10.1007/s00438-016-1203-2.
60. Mehrtens, F.; Kranz, H.; Bednarek, P.; Weisshaar, B. The Arabidopsis Transcription Factor MYB12 Is a Flavonol-Specific Regulator of Phenylpropanoid Biosynthesis. *Plant Physiology* **2005**, *138*, 1083-1096, doi:10.1104/pp.104.058032.
61. Nakabayashi, R.; Yonekura-Sakakibara, K.; Urano, K.; Suzuki, M.; Yamada, Y.; Nishizawa, T.; Matsuda, F.; Kojima, M.; Sakakibara, H.; Shinozaki, K.; et al. Enhancement of oxidative and drought tolerance in Arabidopsis by overaccumulation of antioxidant flavonoids. *The Plant Journal* **2014**, *77*, 367-379, doi:https://doi.org/10.1111/tpj.12388.
62. Reyes, J.L.; Chua, N.-H. ABA induction of miR159 controls transcript levels of two MYB factors during Arabidopsis seed germination. *The Plant Journal* **2007**, *49*, 592-606, doi:https://doi.org/10.1111/j.1365-313X.2006.02980.x.
63. Cui, M.H.; Yoo, K.S.; Hyoung, S.; Nguyen, H.T.K.; Kim, Y.Y.; Kim, H.J.; Ok, S.H.; Yoo, S.D.; Shin, J.S. An Arabidopsis R2R3-MYB transcription factor, AtMYB20, negatively regulates type 2C serine/threonine protein phosphatases to enhance salt tolerance. *FEBS Letters* **2013**, *587*, 1773-1778, doi:https://doi.org/10.1016/j.febslet.2013.04.028.
64. Gao, S.; Zhang, Y.L.; Yang, L.; Song, J.B.; Yang, Z.M. AtMYB20 is negatively involved in plant adaptive response to drought stress. *Plant and Soil* **2014**, *376*, 433-443, doi:10.1007/s11104-013-1992-6.
65. Sun, Y.; Zhao, J.; Li, X.; Li, Y. E2 conjugases UBC1 and UBC2 regulate MYB42-mediated SOS pathway in response to salt stress in Arabidopsis. *New Phytologist* **2020**, *227*, 455-472, doi:https://doi.org/10.1111/nph.16538.
66. Yan, C.; Chai, J.; Zheng, Q.; Li, S.; Li, M.; Wang, X.; Zhang, Q.; Wang, X.; Zhu, Z. The MYB transcription factors directly mediate abscisic acid signals in response to abiotic stress. *Plant Science* **2026**, *363*, 112890, doi:https://doi.org/10.1016/j.plantsci.2025.112890.
67. Chang, Y.K.; Shi, M.M.; Wang, X.; Cheng, H.; Zhang, J.L.; Liu, H.R.; Wu, H.R.; Ou, X.B.; Yu, K.; Zhang, X.B.; et al. A CRY1-HY5-MYB signaling cascade fine-tunes guard cell reactive oxygen species levels and triggers stomatal opening. *PLANT CELL* **2025**, *37*, doi:10.1093/plcell/koaf064.
68. Li, X.; Lu, J.; Zhu, X.; Dong, Y.; Liu, Y.; Chu, S.; Xiong, E.; Zheng, X.; Jiao, Y. AtMYBS1 negatively regulates heat tolerance by directly repressing the expression of MAX1 required for strigolactone biosynthesis in Arabidopsis. *Plant Communications* **2023**, *4*, doi:10.1016/j.xplc.2023.100675.
69. Wu, Q.; Tao, Y.; Huang, J.; Liu, Y.S.; Yang, X.Z.; Jing, H.K.; Shen, R.F.; Zhu, X.F. The MYB transcription factor MYB103 acts upstream of TRICHOME BIREFRINGENCE-LIKE27 in regulating aluminum sensitivity by modulating the O-acetylation level of cell wall xyloglucan in Arabidopsis thaliana. *The Plant Journal* **2022**, *111*, 529-545, doi:https://doi.org/10.1111/tpj.15837.
70. Kim, S.H.; Lam, P.Y.; Lee, M.-H.; Jeon, H.S.; Tobimatsu, Y.; Park, O.K. The Arabidopsis R2R3 MYB Transcription Factor MYB15 Is a Key Regulator of Lignin Biosynthesis in Effector-Triggered Immunity. *Frontiers in Plant Science* **2020**, *Volume 11 - 2020*.
71. Beathard, C.; Mooney, S.; Al-Saharin, R.; Goyer, A.; Hellmann, H. Characterization of Arabidopsis thaliana R2R3 S23 MYB Transcription Factors as Novel Targets of the Ubiquitin Proteasome-Pathway and Regulators of Salt Stress and Abscisic Acid Response. *Frontiers in Plant Science* **2021**, *Volume 12 - 2021*.
72. Li, Y.; Tian, B.; Wang, Y.; Wang, J.; Zhang, H.; Wang, L.; Sun, G.; Yu, Y.; Zhang, H. The Transcription Factor MYB37 Positively Regulates Photosynthetic Inhibition and Oxidative Damage in Arabidopsis Leaves Under Salt Stress. *Frontiers in Plant*

Science **2022**, *Volume 13 - 2022*.

73. Li, X.; Zhong, M.; Qu, L.; Yang, J.; Liu, X.; Zhao, Q.; Liu, X.; Zhao, X. AtMYB32 regulates the ABA response by targeting ABI3, ABI4 and ABI5 and the drought response by targeting CBF4 in Arabidopsis. *Plant Science* **2021**, *310*, 110983, doi:https://doi.org/10.1016/j.plantsci.2021.110983.
74. Zhao, H.; Jiang, J.; Shen, M.; Zhang, Y.; Zhang, Y.; Liu, H.; Zhou, H.; Zheng, Y. The transcription factor MYB30 promotes iron homeostasis by maintaining the stability of the FIT transcription factor. *Plant Cell* **2025**, *37*, doi:10.1093/plcell/koaf090.
75. Xiao, Q.; Wang, Y.; Du, J.; Li, H.; Wei, B.; Wang, Y.; Li, Y.; Yu, G.; Liu, H.; Zhang, J.; et al. ZmMYB14 is an important transcription factor involved in the regulation of the activity of the ZmBT1 promoter in starch biosynthesis in maize. *The FEBS Journal* **2017**, *284*, 3079-3099, doi:https://doi.org/10.1111/febs.14179.
76. Han, J.N.; Li, R.; Zhang, Z.; Liu, S.Y.; Liu, Q.Q.; Xu, Z.N.; Zhou, Z.Q.; Lu, X.; Shangguan, X.C.; Zhou, T.F.; et al. Genome-wide association and co-expression uncovered ZmMYB71 controls kernel starch content in maize. *JOURNAL OF INTEGRATIVE AGRICULTURE* **2025**, *24*, 4496-4514, doi:10.1016/j.jia.2024.03.013.
77. Zhang, X.; Li, H.Y.; Zhao, C.Y.; Wu, Y.; Lu, X.D. ZmMYB92 modulates secondary wall cellulose synthesis in maize. *PLANT JOURNAL* **2025**, *122*, doi:10.1111/tpj.70296.
78. Li, Q.Z.; Wang, J.H.; Zhou, A.; Guo, Y.X.; Li, J.T.; Ji, Y.; Cheng, L.; Yu, C.Y.; Wang, L.; Wu, C.Y.; et al. ZmMYB155 is involved in starch synthesis and basal endosperm transfer layer development in maize. *PLANT CELL REPORTS* **2025**, *44*, doi:10.1007/s00299-025-03569-9.
79. Long, T.D.; Wang, Y.Y.; Liu, Z.; Wang, Y.B.; Mao, C.Q.; Wang, D.N.; Qin, A.Y.; Liao, Q.; Yang, J.; Fan, X.J.; et al. ZmMYB127 Modulates Maize Kernel Texture and Size by Integrating the Synthesis of Starch, Zein Proteins and Auxin. *PLANT BIOTECHNOLOGY JOURNAL* **2026**, *24*, 810-827, doi:10.1111/pbi.70384.
80. Shi, J.; Li, Z.Q.; Wang, Z.Y.; Pan, S.X.; Wu, X.; Wang, X.; Ye, Y.F.; Xu, Z.P.; He, J.J.; Zhang, Z.Y. ZmMYB127 controls maize endosperm filling via dual-transcriptional regulation to improve grain yield and quality. *NATURE PLANTS* **2026**, *12*, doi:10.1038/s41477-026-02238-3.
81. Chen, Z.; Teng, S.; Liu, D.; Chang, Y.; Zhang, L.; Cui, X.; Wu, J.; Ai, P.; Sun, X.; Lu, T. RLM1, encoding an R2R3 MYB transcription factor, regulates the development of secondary cell wall in rice. *Frontiers in Plant Science* **2022**, *13*, 905111.
82. Zou, T.; Wang, X.; Sun, T.; Rong, H.; Wu, L.; Deng, J.; Guo, T.; Wang, H.; Wang, J.; Huang, M.A.-O. MYB Transcription Factor OsC1(PLSr) Involves the Regulation of Purple Leaf Sheath in Rice. LID - 10.3390/ijms24076655 [doi] LID - 6655. *Int J Mol Sci. 24*, 6655. doi: 6610.3390/ijms24076655.
83. Zheng, J.; Wu, H.; Zhu, H.; Huang, C.; Liu, C.; Chang, Y.; Kong, Z.; Zhou, Z.; Wang, G.; Lin, Y.; et al. Determining factors, regulation system, and domestication of anthocyanin biosynthesis in rice leaves. *New Phytologist* **2019**, *223*, 705-721, doi:https://doi.org/10.1111/nph.15807.
84. Yin, M.M.; Wei, C.H.; Du, H.M.; Lyu, T.; Luo, F.; Zhang, W.F.; Zhou, X.L.; Wang, C.B.S.; Chen, L.J.; Lee, D.S. Comprehensive analysis of R2R3-MYB transcription factors reveals OsMYB1 as a key regulator of anthocyanin biosynthesis in rice. *PLANT SCIENCE* **2025**, *360*, doi:10.1016/j.plantsci.2025.112732.
85. Zhou, J.; Lee, C.; Zhong, R.; Ye, Z.-H. MYB58 and MYB63 Are Transcriptional Activators of the Lignin Biosynthetic Pathway during Secondary Cell Wall Formation in Arabidopsis *The Plant Cell* **2009**, *21*, 248-266, doi:10.1105/tpc.108.063321.
86. Gigolashvili, T.; Berger, B.; Mock, H.-P.; Müller, C.; Weisshaar, B.; Flügge, U.-I. The transcription factor HIG1/MYB51 regulates indolic glucosinolate biosynthesis in Arabidopsis thaliana. *The Plant Journal* **2007**, *50*, 886-901, doi:https://doi.org/10.1111/j.1365-313X.2007.03099.x.
87. Zhong, R.; Lee, C.; Zhou, J.; McCarthy, R.L.; Ye, Z.-H. A Battery of Transcription Factors Involved in the Regulation of Secondary Cell Wall Biosynthesis in Arabidopsis. *The Plant Cell* **2008**, *20*, 2763-2782, doi:10.1105/tpc.108.061325.
